# Discrimination of 14 olive cultivars using morphological analysis and machine learning algorithms

**DOI:** 10.3389/fpls.2024.1441737

**Published:** 2024-08-08

**Authors:** Konstantinos N. Blazakis, Danil Stupichev, Maria Kosma, Mohamad Ali Hassan El Chami, Anastasia Apodiakou, George Kostelenos, Panagiotis Kalaitzis

**Affiliations:** ^1^ Department of Horticultural Genetics and Biotechnology, Mediterranean Agronomic Institute of Chania (MAICh), Chania, Greece; ^2^ Kostelenos Olive Nurseries, Poros, Greece

**Keywords:** olive cultivar identification, morphological analysis, machine learning, image analysis, olive fruit, olive leaf, olive endocarp

## Abstract

Traditional morphological analysis is a widely employed tool for the identification and discrimination of olive germplasm by using morphological markers which are monitored by subjective manual measurements that are labor intensive and time-consuming. Alternatively, an automated methodology can quantify the geometrical features of fruits, leaves and endocarps with high accuracy and efficiency in order to define their morphological characteristics. In this study, 24 characteristics for fruits, 16 for leaves and 25 for endocarps were determined and used in an automated way with basic classifiers combined with a meta-classsifier approach. This resulted to the discrimination of 14 olive cultivars utilizing data obtained from two consecutive olive growing periods. The cultivar classification algorithms were based on machine learning techniques. The 95% accuracy rate of the meta-classifier approach indicated that was an efficient tool to discriminate olive cultivars. The contribution of each morphological feature to cultivar discrimination was quantified, and the significance of each one was automatically detected in a quantitative way. The higher the contribution of each feature, the higher the significance for cultivar discrimination. The identification of most cultivars was guided by the features of both endocarps and fruits, while those of leaves were only efficient to identify the Kalamon cultivar. The combined use of morphological features of three olive organs might have an additive effect leading to higher capacity for discrimination of cultivars. The proposed methodology might be considered a phenomics tool for olive cultivar identification and discrimination in a wide range of applications including breeding.

## 1 Introduction

Olive (*Olea europaea* L. subsp. *europaea* var. *europaea*) is an important fruit tree crop in the Mediterranean Basin while its cultivation has expanded across the rest of the world, including the United States of America, Oceania, South Africa and Asia (Torres et al., 2017; Koubouris et al., 2019; Mousavi et al., 2019). Olive oil originating in the Mediterranean region accounts for more than 90% of global production and has significant socioeconomic importance for the European Union, since Spain, Italy, Greece and Portugal produce approximately 75% of the world’s olive oil supply (Paredes et al., 2019). The ability to identify and discriminate olive cultivars is important for the efficient management and exploitation of the available olive genetic resources and for breeding programs (Beiki et al., 2012; De Ollas et al., 2019). The identification, cataloguing and exploitation of germplasm collections comprising cultivars and accessions is performed by using morphological and molecular markers (Rallo et al., 2018).

Molecular markers and new, advanced biotechnological platforms have been used for genetic diversity assessment and cultivar discrimination (D’Imperio et al., 2011; Muleo et al., 2016; Sebastiani and Busconi, 2017; Zhu et al., 2019; Gomez-Rodrıguez et al., 2021) while morphological markers continue to constitute the main approach for describing and discriminating olive germplasm, despite limitations such as the variability of environmental conditions, the age of trees, agronomical practices and phenological stage of trees (Trujillo et al., 2014; Blazakis et al., 2017).

Molecular markers and new, advanced biotechnological platforms have been used for genetic diversity assessment and cultivar discrimination (Diaz et al., 2004; D’Imperio et al., 2011; Muleo et al., 2016; Sebastiani and Busconi, 2017; Zhu et al., 2019; Gomez-Rodrıguez et al., 2021). However, morphological markers continue to constitute the main approach for describing and discriminating olive germplasm, despite limitations such as the variability of environmental conditions, the age of trees, agronomical practices, and the phenological stage of trees (Trujillo et al., 2014; Blazakis et al., 2017). Most morphological studies are based on a simplified scheme that has been adopted by the International Union for the Protection of New Varieties of Plants (UPOV) which focuses on the morphological characteristics of leaves, fruits and endocarps (Trujillo et al., 2014). These characteristics have been widely used for descriptive purposes to distinguish olive cultivars (Barranco et al., 2000; Trujillo et al., 2014; Beyaz et al., 2017; Blazakis et al., 2017; Rallo et al., 2018; Koubouris et al., 2019). Currently, most morphological characterization of olive organs is performed by either time-consuming, labor-intensive, manual measurements or by one of several stand-alone software-based methodologies (Riquelme et al., 2008; Rodríguez et al., 2010; Orrù et al., 2013; Beyaz et al., 2017).

Despite the extensive use of morphological characteristics of olive fruits, leaves and endocarps for the identification of cultivars, there is a lack of automated methodologies to further assist in the development of this field. Working in this direction, (Blazakis et al., 2017) an integrated image-based tool on automated methodology was developed in order to describe olive fruit, leaf and endocarp morphologies. The methodology quantifies many features of these organs based on strictly mathematical morphological parameters and provides accurate, objective numerical measurements of the olive organ morphology attributes in a more robust and efficient way.

Currently, there are several olive cultivar identification methods that use chemical and genetic fingerprinting techniques. However, these methods require a high level of specialization, specific expensive infrastructure, and laboratory work. In contrast, user-friendly image-based methodologies could enable fast, accurate identification of olive cultivars that could be valuable for farmers, food inspection authorities or researchers (Beiki et al., 2012; Trujillo et al., 2014; Ponce et al., 2019; Gomes et al., 2020; Khadivi et al., 2022; Gago et al., 2024). The effectiveness and accuracy of image-based methodologies for morphological characterization of different crop species is usually linked to cultivar discrimination and classification (Fuentes et al., 2018; Ishikawa et al., 2018; Wäldchen et al., 2018; Dheer and Singh, 2019). Traditionally, a rapid cultivar identification is a non-automated process involving visual inspection: the user tries to identify the organ characteristics that will be considered the discriminating keys for each cultivar (Wäldchen et al., 2018). Discriminating morphological characteristics of fruits, leaves, endocarps or other organs is commonly used for a quick cultivar identification based on appearance, but visual observations require experience and sometimes appear to be very subjective, inconsistent and inaccurate (Grinblat et al., 2016; Fuentes et al., 2018; Wäldchen and Mäder, 2018). Automated methodologies for plant cultivar identification based only on morphological characteristics are still in the very early stages (Ishikawa et al., 2018; Miho et al., 2024).

This report focusses on the design of an automated methodology for olive cultivar discrimination based on the calculation of different morphological features of fruits, leaves and endocarps through geometrical feature extraction and cultivar classification. The cultivar classification algorithms are based on established machine learning techniques, while the morphological analysis was based on a previously developed methodology (Blazakis et al., 2017). This new automated methodology takes a further step in the development of an integrated automated tool to characterize, identify and discriminate a large set of olive cultivars using machine learning approaches. The relative contribution of each morphological feature for olive cultivar discrimination was also determined in a quantitative way.

## 2 Materials and methods

### 2.1 Plant material

Fourteen Greek and international olive cultivars were discriminated in this study: Arbequina, Arbosana, Asprolia Alexandroupolis, Kalamon, Karidolia Chalkidikis, Koroneiki, Kothreiki, Koutsourelia, Mastoidis Gigas, Mavrolia Serron, Megaron, Ntopia Atsiholou, Thiaki and Tragolia. All fruit, leaf and endocarp samples of the olive cultivars were collected in 2016 and 2017 from the olive germplasm collection of the commercial nursery “Kostelenos” in Poros, Trizinia in Greece. All the trees were grown under identical conditions, and the fruit samples were collected at the breaker stage. Fruits were collected from the middle part of one year-old shoots around the canopy at approximately 1.5 m height from the ground. They were collected from fruit-bearing branches avoiding irregular fruits and taking into account the fruit load of each tree. Moreover, mature, healthy leaves were collected from the most representative one year-old shoots located on the southern part of the tree. Finally, the endocarps were extracted from the sample fruits, and the pulp was removed by a coarse fabric. All kernels were soaked in 10% bleach for 5 minutes and stored in a dry place for later usage. At least 100 samples from fruits, leaves or endocarps were used for the morphological analysis and the classification algorithms.

### 2.2 Morphological analysis

To generate the imaging data for the olive samples, we followed the methodology described in (Blazakis et al., 2017). The morphological analysis of the fruit and endocarp samples was performed using the imaging positions adopted by UPOV and the International Olive Council (IOC). However, for the development of the classification algorithms we used all the numerical values corresponding to both positions of fruits and endocarps. A meopta copy imaging stand was used to create the imaging data of fruits and endocarps. All the samples were placed on a 2mm-thick piece of elevated glass to eliminate shadows, and the camera was installed above them, on a fixed solid arm. An HP DeskJet Ink Advantage 3636 scanner was used to scan the leaves at a resolution of at least 600 dots per inch (dpi). All the photographs were saved as jpeg or png files, and a scaler was placed next to them.

Next, we separated the items in an image from their background (a process known as segmentation). This resulted in a binary image of each shape. The morphological analysis of olive fruits, leaves and endocarps was performed using OliveID, a set of state-of-the-art automatic algorithms for object contour extraction from imaging data that was developed in MATLAB (The Mathworks Inc., Natick, MA, USA) (Guide, 1998; Blazakis et al., 2017). OliveID is a computational methodology for olive morphological analysis that identifies various geometrical characteristics which are assigned to different morphological traits. The outcome of the algorithm is the representation of each shape by a discrete sequence with all its boundary points that enables us to quantitatively and qualitatively analyze the morphology of the olives, leaves and endocarps of each cultivar. For the morphological analysis of fruit, we used 24 parameters that describe fruit morphological characteristics, while we used 16 for the leaf. Finally, for the stone’s morphology description in two positions (A and B) we used 22 morphological characters. All the morphological characters were purely mathematically defined, and we refer to (Blazakis et al., 2017) and the references therein, for further details.

### 2.3 Data processing and analysis

The statistical analysis of datasets and the creation and study of classifiers were implemented in a Jupyter Notebook in Python (Kluyver et al., 2016). Principal component analysis (PCA) was executed to visualize the initial classification of the olive cultivars based on quantitative data retrieved from the analysis that corresponds to the morphological traits of olive fruits, leaves and endocarps. It was implemented using the scikit-learn library (Pedregosa et al., 2011) in Python. For a more comprehensive view of the data set collected and calculated during the two consecutive years, split violin plots were built which allowed a direct comparison between the years. Violin plots can graphically represent the data distribution of a set of data by combining a box plot and a rotated density plot. By inspecting the shape of the violin plot representing the density estimate of the data points, regions with a higher frequency of particular values can easily be highlighted. Within a split violin plot, the left side represents the numerical data regarding the specific morphological characteristic relative to the first year, while the right side is for data extracted from the next year’s morphological analysis.

### 2.4 Classification algorithm

The ability to explain in understandable terms why a machine learning model makes a certain classification is becoming immensely important, as it ensures trust and transparency in the decision-making process of the model. Therefore, in order to explore the morphological features’ importance in the classification process, a general model-agnostic method for model interpretation, Shapley values, was used. Shapley values can provide accurate explanations, as they assign each morphological feature an importance value for olive cultivar prediction and determination (Kumar et al., 2020; Wang et al., 2021). It was implemented using the scikit-learn library (Pedregosa et al., 2011) in Python.

### 2.5 Machine learning classifier algorithms for olive cultivar identification

Supervised and unsupervised machine learning techniques provide a powerful tool for agriculture due to their wide range of applications, such as detection of crop disease, crop management and plant phenotyping (Rehman et al., 2019). The task of discriminating olive cultivars based on morphological parameters of olive fruits, leaves and endocarps relies on a data mining problem in which several classification supervised learning methods were tested for an appropriate classification model (Gomes et al., 2020).

The proposed methodology uses classifiers of two different types:

- basic classifiers that determine the cultivar by using the quantitative data revealed by the morphological analysis of either fruit, leaf, or endocarp;
- meta-classifiers that determine the cultivar by using all data from the morphological analysis of olive fruit, leaf and endocarp.

The Random Forest Classifier (Breiman, 2001), k-Nearest Neighbor (Venables and Ripley, 2002) and Support Vector Classifier (Boser et al., 1992) and the scikit-learn library (Pedregosa et al., 2011) were used as basic classifiers for olive fruits, leaves, and endocarps. Of all the classification algorithms tested, the XGBClassifier had an advantage due to the high accuracy of the classifier analysis.

In order to perform the olive cultivar identification based on the morphological analysis of a plant’s organs, we use the stacking method (Dou et al., 2020), in which not raw data are used for training, but probability matrices from the basic classifiers. The meta-classifier learning process can be described as follows (**Supplementary Figure 1**):

1. Initially, the entire dataset is divided into two different sets. 9/10 of the whole dataset is used for training; and the other 1/10 is used at the end to test the meta-classifier.
2. Then the first set which was intended for training of the algorithm is sub-divided into 5 parts. 4/5 is used to train the three individual basic classifiers of fruits, leaves and endocarps.
3. After training, the remaining 1/5 of the data is used as input for the basic classifiers, and the result of the classification is 3 matrices of the probability of attribution to the cultivar by fruit, leaf and endocarp. Obtained matrices of the probabilities are used as a training sample for the meta-classifier.
4. The meta-classifier is trained on the new data set, and the cross-validation algorithm is performed to obtain the metric of such classification.

## 3 Results

### 3.1 Morphological characterization

**Figure 1:**
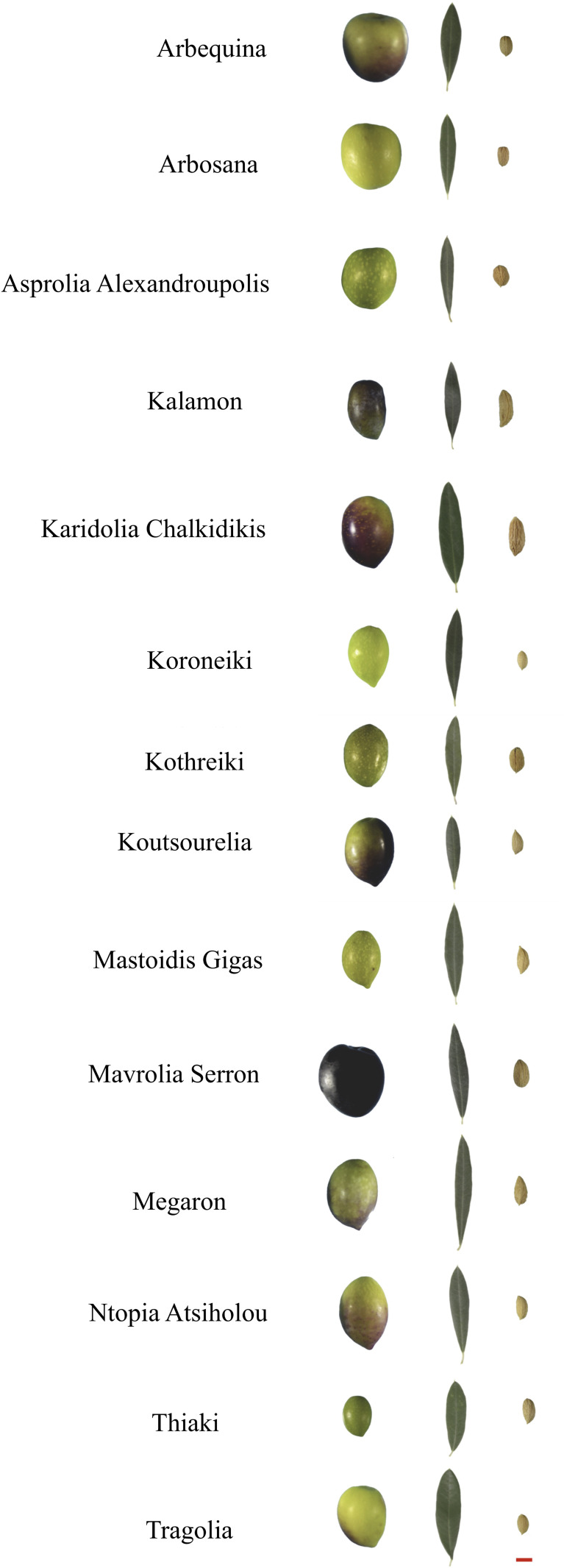
Morphological features of olive cultivars. Fruits, leaves and endocarps of Arbequina, Arbosana, Asprolia Alexandroupolis, Kalamon, Karidolia Chalkidikis, Koroneiki, Kothreiki, Koutsourelia, Mastoidis Gigas, Mavrolia Serron, Megaron, Ntopia Atsiholou, Thiaki, Tragolia are presented with a similar scale. The images were modified to indicate the actual sizes for comparative purposes. The red line represents 1cm.

A group of 14 olive cultivars was characterized at the morphological level and a wide range of diversity was determined (**Figure 1**). This group was comprised of 12 Greek cultivars, Asprolia Alexandroupolis, Kalamon, Karidolia Chalkidikis, Koroneiki, Kothreiki, Koutsourelia, Mastoidis Gigas, Mavrolia Serron, Megaron, Ntopia Atsiholou, Thiaki, and Tragolia and two Spanish cultivars, Arbosana and Arbequina (**Figure 1**). The indicative fruit, leaf and endocarp samples were acquired from the first growing period (**Figure 1**). According to visual judgement, Karidolia-Chalkidikis and Mavrolia-Serron have larger fruits. The leaf morphology of Kalamon was also distinguished compared to the other cultivars, as it is substantially larger (**Supplementary Figure 2**). The endocarps of Karidolia-Chalkidikis and Mavrolia-Serron were the largest in size.

**Figure 2:**
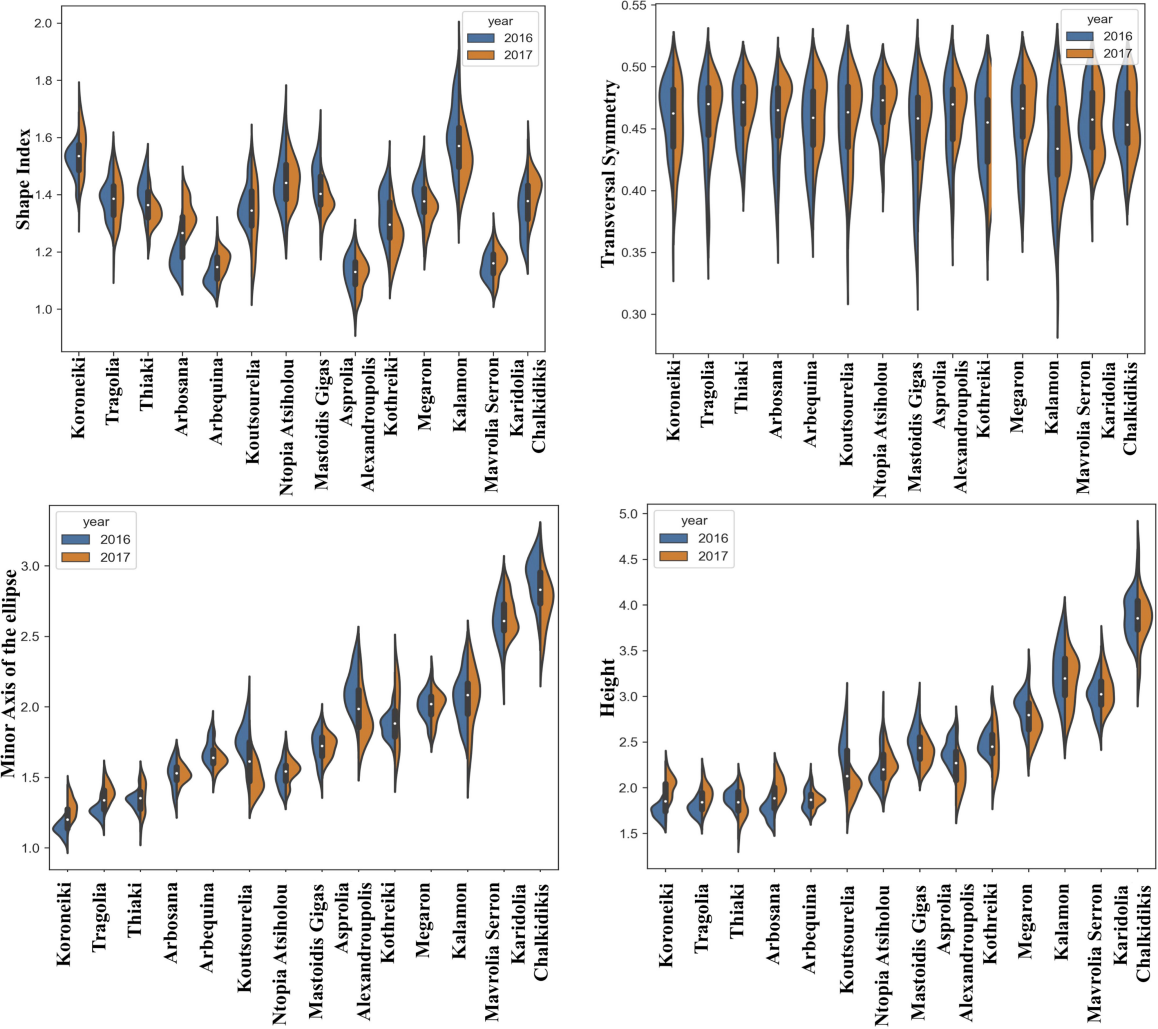
Morphological analysis. Violin graphs for fruits. Violin plots representing trait variation in two consecutive years. Each plot shows the distribution of data from the minimum to the maximum level, with white inner dot showing the data median for 14 cultivars. The black boxplots represent the lower and upper limits of the first and third quartiles. The outliers were removed. The horizontal width of the violin depends on the data density.

**Figure 3:**
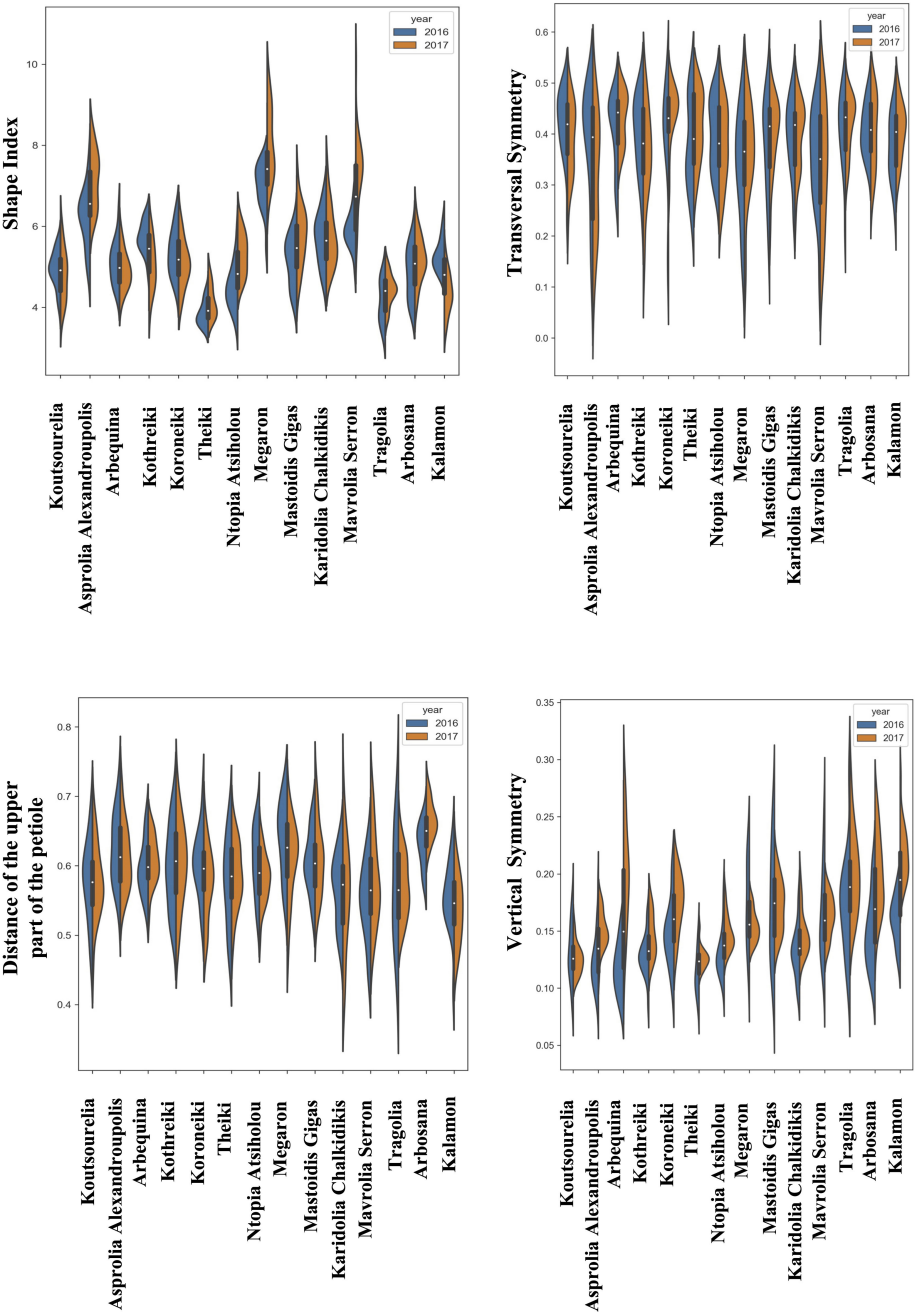
Morphological analysis. Violin graphs for leaves. Violin plots representing trait variation in two consecutive years. Each plot shows the distribution of data from the minimum to the maximum level, with white inner dot showing the data median for 14 cultivars. The black boxplots represent the lower and upper limits of the first and third quartiles. The outliers were removed. The horizontal width of the violin depends on the data density.

**Figure 4:**
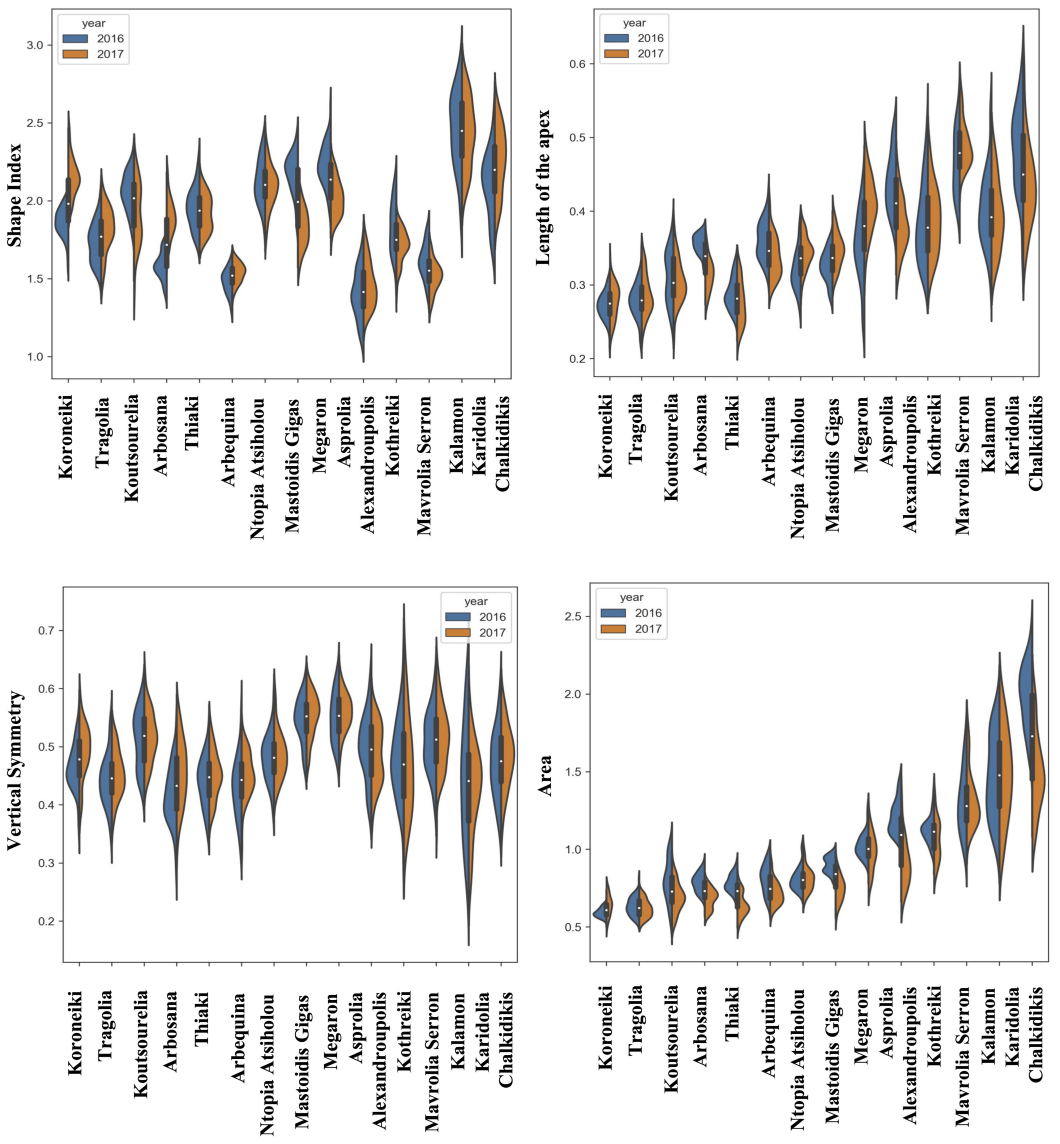
Morphological analysis. Violin graphs for endocarps. Violin plots representing trait variation in two consecutive years. Each plot shows the distribution of data from the minimum to the maximum level, with white inner dot showing the data median for 14 cultivars. The black boxplots represent the lower and upper limits of the first and third quartiles. The outliers were removed. The horizontal width of the violin depends on the data density.

The quantitative data of morphological features were acquired from the consecutive growing periods of 2016 and 2017. The distribution of the quantitative morphological features was visualized using split violin plots to take into consideration the variation due to climatic conditions, which showed the kernel density estimation of the data as well as the median and the upper and lower quartiles. **Figures 2**–**4** show the fruit, leaf and endocarp split violin plots for the most remarkable morphological features, respectively. All the data are presented in the **Supplementary Figure 2**.

The shape of fruits appeared to be similar for most of the cultivars in the two consecutive growing periods, with the exception of Arbosana, which showed a different shape distribution each year. The fruit shape index indicated that Koroneiki and Kalamon had more elongated fruits, whereas Asprolia-Alexandroupolis and Mavrolia-Serron had more spherical fruits. Furthermore, the Koroneiki, Tragolia and Thiaki fruits had a more pointed apex than Mavrolia-Serron and Karidolia-Alexandroupolis fruits, in which the apex curvature was close to zero. The height and length of the minor axis of a fitted ellipse to the fruits followed the same tendency in the two growing seasons.

The leaves of Megaron, Asprolia-Alexandroupolis and Mavrolia-Serron were the only ones with a lanceolate shape, while the maximum transverse diameter of blades appeared to be medium sized in most cultivars. Kalamon appeared to be the only cultivar with a wide leaf blade, while Koutsourelia, Asprolia-Alexandroupolis and Megaron cultivars had narrow leaves. The thickness of the upper part of the petiole was also determined quantitatively, with Asprolia-Alexandroupolis and Arbequina displaying contrasting profiles in two consecutive years. The vertical symmetry of the leaf clearly illustrated that the maximum transverse diameter was located towards the apex, and this remained stable in time.

**Figure 5:**
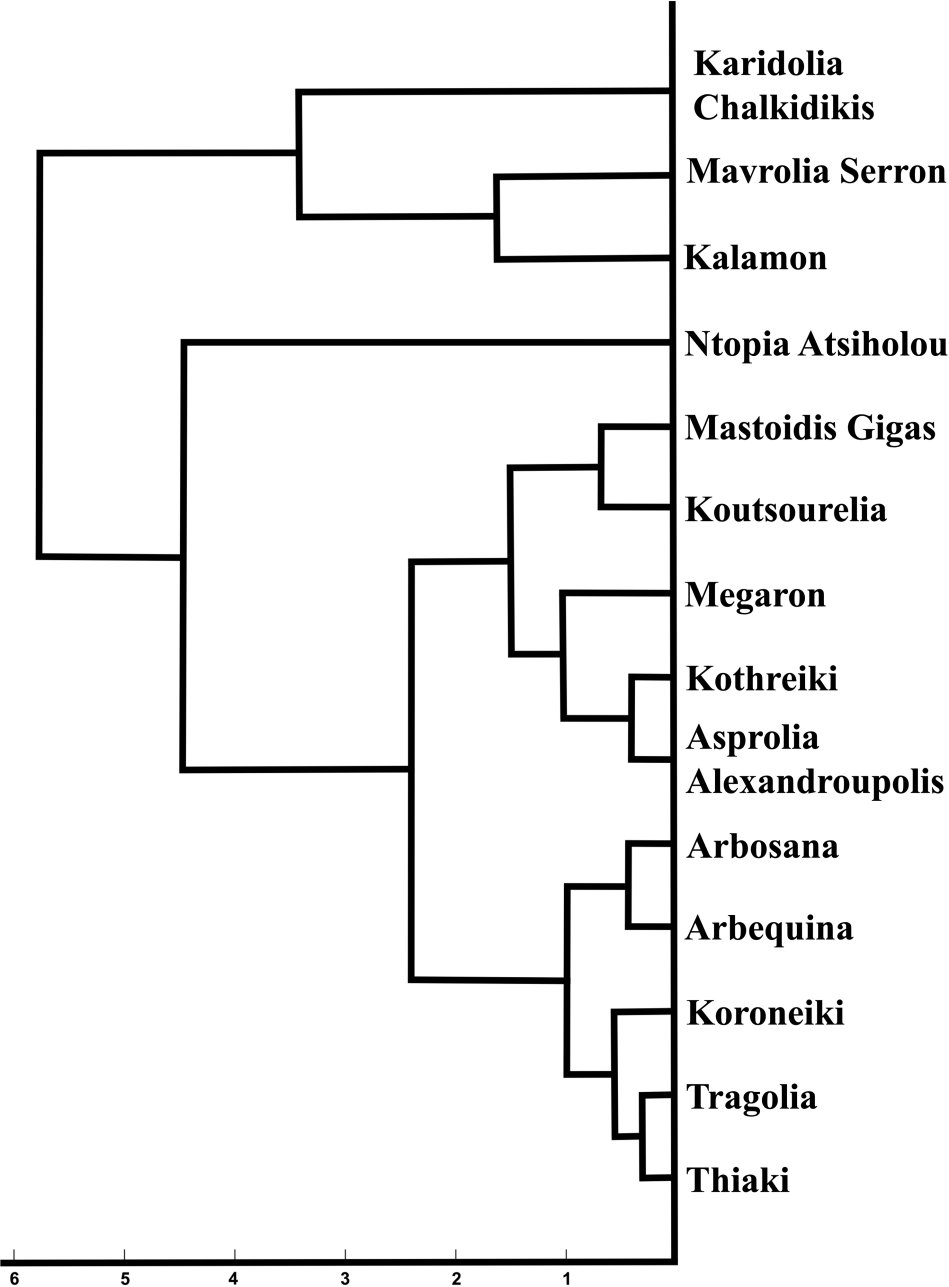
Dendrogram indicating the clustering of the 14 cultivars based on the entire set of morphological characteristics.

The most striking observation regarding the endocarps was that the kernel density estimation of the morphological data remained stable between the two years of growth in most of the cultivars for all the phenotypic characteristics, according to the split violin plots of the endocarp morphological analysis. Koroneiki and Tragolia had small endocarps, while Karidolia-Chalkidikis and Kalamon had larger ones. The Tragolia endocarps had an ovoid shape, whereas Koroneiki and Koutsourelia were more elliptic based on the shape index. The endocarp shape of Kalamon and Karidolia-Chalkidikis appeared to be elongated. According to the shape index, cultivars were clustered in two groups: one with an ovoid shape, and another with an elliptic shape. This methodology also successfully detected the endocarp apex length. Comparing the area of the apex curve and the endocarp size in both years, the cultivars of Mastoidis and Koutsourelia had a mucro present, whereas it was absent in Kothreiki. **Figure 5** shows a dendrogram indicating the clustering of the 14 cultivars based on the entire set of morphological characteristics. Four main groups were identified, all but one consisting of three to five cultivars. Only the cultivar of Ntopia Atsiholou is grouped distantly from the other clusters. It is interesting that Thiaki and Tragolia showed higher similarity to Koroneiki compared to the other cultivars. Moreover, the two Spanish cultivars, Arbequina and Arbosana, are grouped together in one sub cluster, separately from the Greek cultivars. Finally, the cultivars of Kalamon, Mavrolia Serron and Karidolia Chalkidikis form the cluster with the largest fruits.

### 3.2 The effect of environment conditions on morphological parameters

Wide variation in the morphological characteristics was detected due to variations in the environmental conditions in the two sampling years (**Supplementary Table 1**). In fruits, higher variability was observed in the first growing period compared to the second. The size of the fruit nipple showed the greatest variability while the nipple index (presence or absence of the nipple) the lowest (**Supplementary Table 1**). Kothreiki and Mastoidis showed the highest variation among all cultivars in fruit area in 2017 and 2016, 0.20 and 0.18, respectively. The fruit shape index was one of the most stable traits over the two growing periods, showing only a 6% coefficient variation (**Supplementary Table 2**), while the fruit area exhibited a higher variability, as expected, compared to the other morphological characteristics of fruit. Moreover, Arbequina showed the highest stability in fruit morphological characteristics, while Koutsourelia was the least stable (**Supplementary Table 2**).

In leaf characteristics, the average curvature of the Kothreiki leaf tip had the highest variability in 2016 (1.74), while the shape index of the Megaron leaf blade had the lowest (0.084). In addition, the leaf petiole of all cultivars was the most variable feature over the two years, whereas the vertical symmetry and the leaf circularity were the most stable (**Supplementary Table 2**).

The observed variation in fruit size among cultivars was accompanied by similar variation in endocarp size. Cultivars with greater fruit size showed also greater endocarp size. The coefficient of variation for all traits of endocarps varies in the lower range of 0.006 to 0.35 (**Supplementary Table 1**). The highest variation appeared in the minimum distance between the transversal diameter and endocarp’s contour of Kalamon in 2016 (CV=0.35), while the lowest appeared in the average circularity of Arbequina endocarps in 2016 (CV=0.006) (**Supplementary Table 1**). The traits related to circularity and the shape index of the endocarp exhibited high stability compared to those related to endocarp area (**Supplementary Tables 1**, **2**).

### 3.3 Principal component analysis

**Figure 6:**
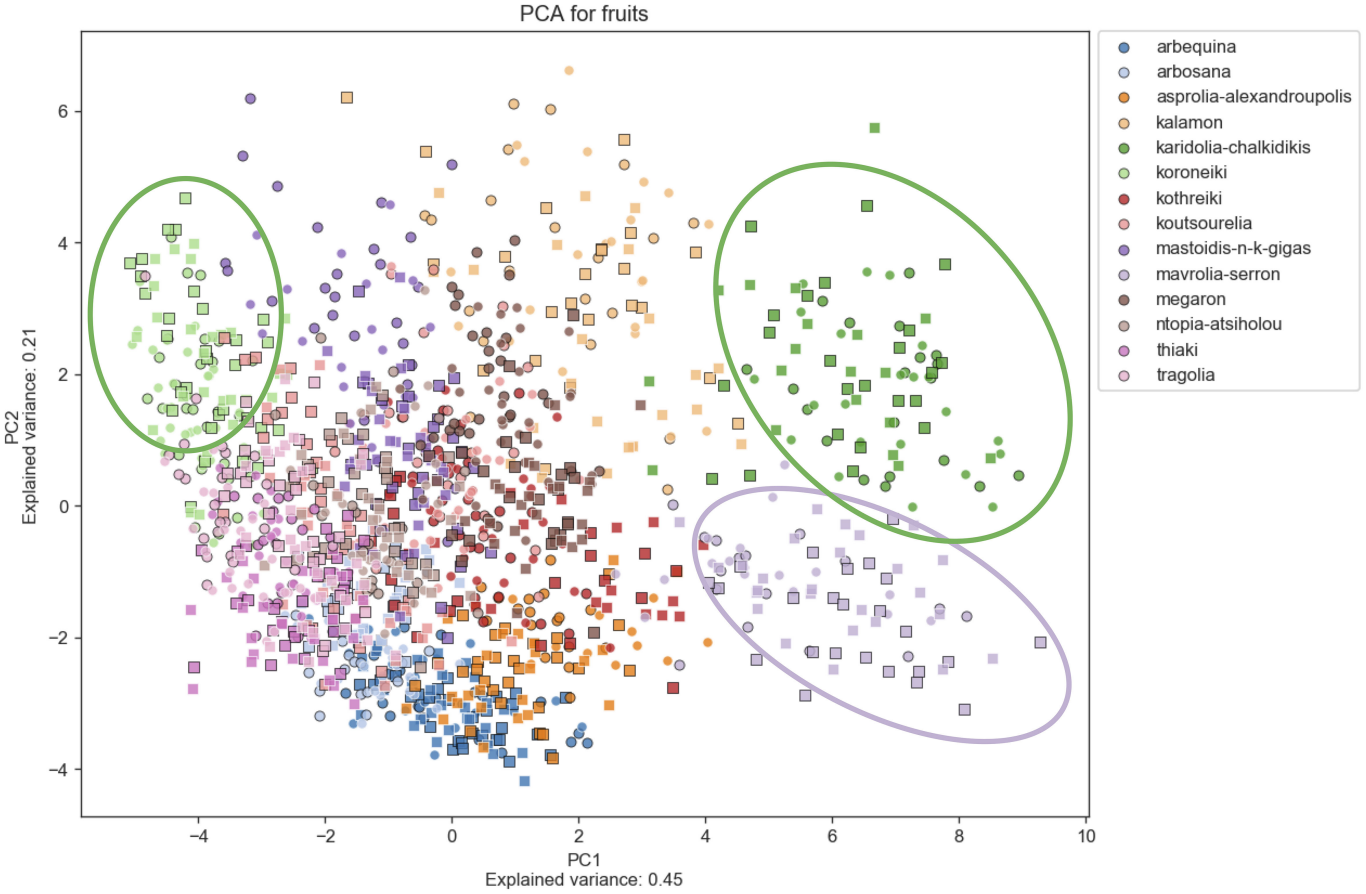
Principal Component Analysis (PCA). PCA for fruits. PCA shows a score plot of the morphological analysis of the fruits of 14 cultivars in two consecutive years. Different colors represent different cultivars. Squares represent the 2016 data, whereas circles represent 2017.

**Figure 7:**
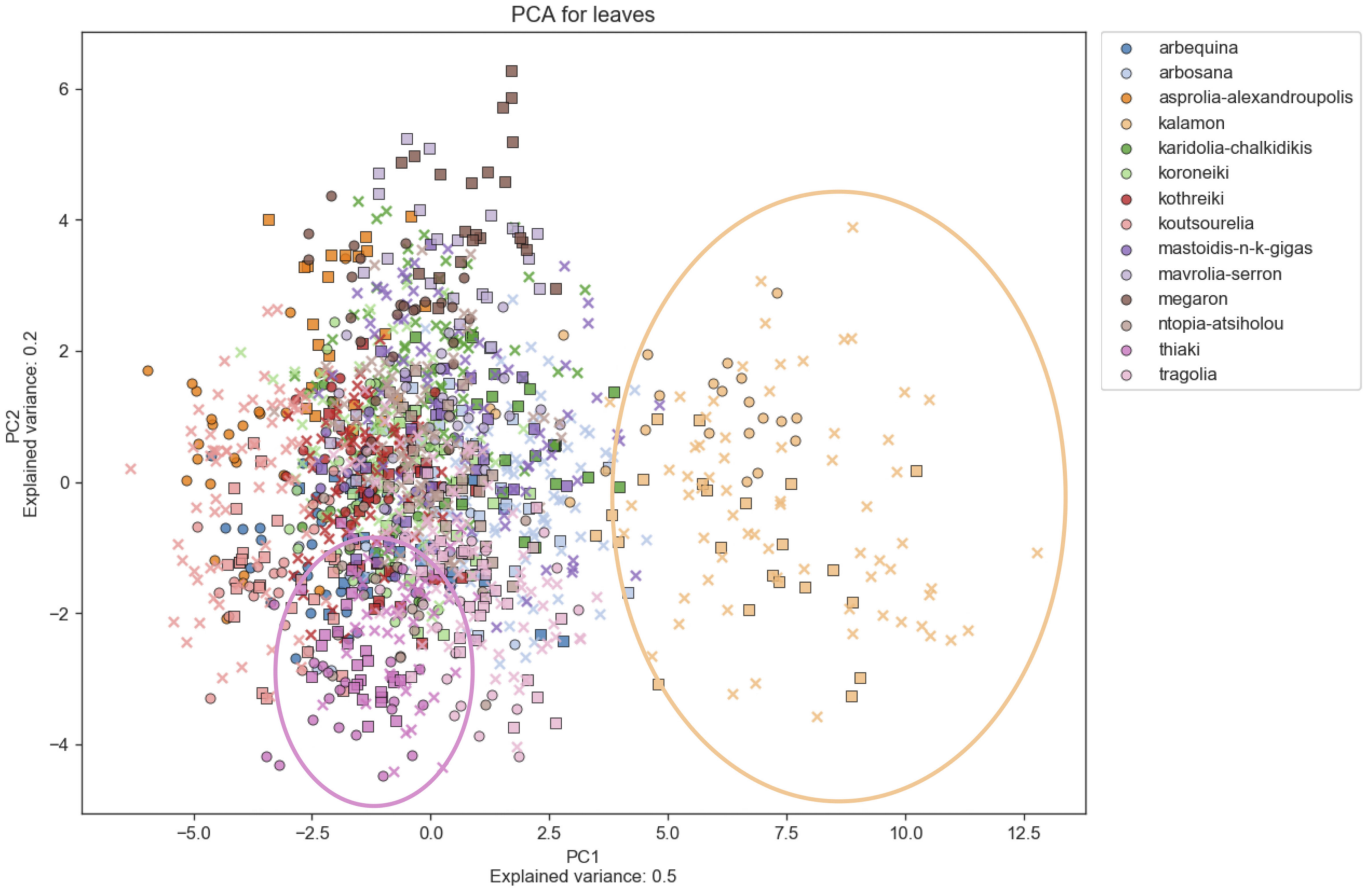
Principal Component Analysis (PCA). PCA for leaves. PCA shows a score plot of the morphological analysis of the leaves of 14 cultivars in two consecutive years. Different colors represent different cultivars. Squares represent the 2016 data, whereas circles represent 2017.

**Figure 8:**
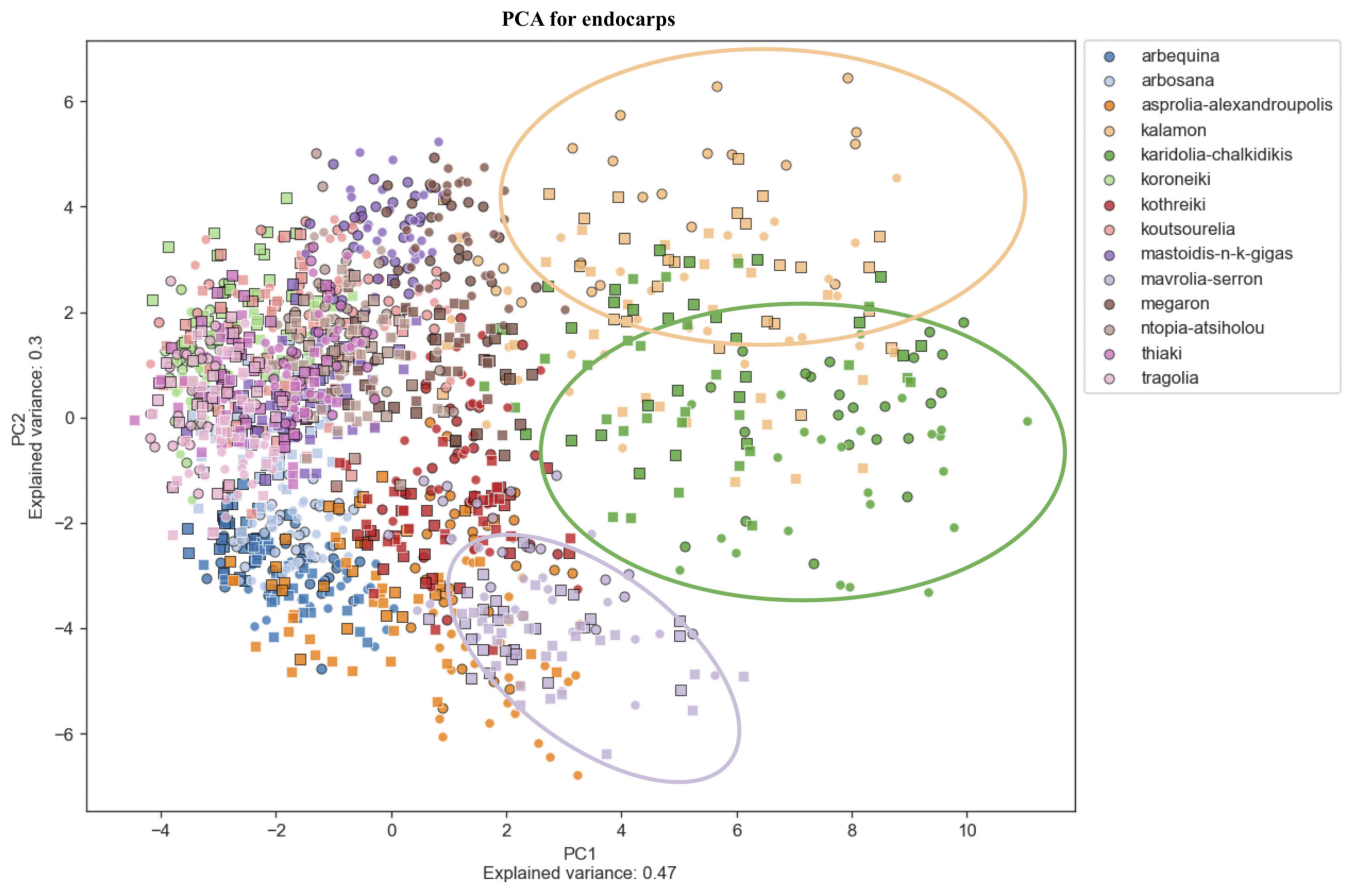
Principal Component Analysis (PCA). PCA for endocarps. PCA shows a score plot of the morphological analysis of the endocarps of 14 cultivars in two consecutive years. Different colors represent different cultivars. Squares represent the 2016 data, whereas circles represent 2017.

Principal Component Analysis (PCA) was used to determine morphological features which differentiate among cultivars, indicating which characteristics are more reliable for discrimination. **Figures 6**–**8** show principal component analysis performed using the numerical data of morphological characteristics of fruits, leaves and endocarps, respectively. The first two components account for approximately 66%, 70%, and 77% of the total variance numerical data, respectively.

PCA revealed significant variation within cultivars which might also be attributed to the differences in environmental conditions between the growing periods. Olive cultivars are dispersed along both principal component axes and are hardly distinguishable from each other, illustrating the complexity of the classification. The two PCA scatter plots of fruits and endocarps illustrate a more even distribution of cultivars, providing higher discriminatory capacity compared to the PCA of leaves. In the leaf PCA, only Kalamon was clearly discriminated from the rest of the cultivars, probably because this cultivar has larger leaves. Karidolia- Chalkidikis in both PCA of fruits and endocarps appears to be more discernible from the other cultivars, which might be attributed to the size of its fruits. Similarly, the fruit size and shape of Kothreiki and Mavrolia-Serron might be responsible for the classification in different groups in the fruit Principal component analysis.

The first principal component of fruits and endocarps was predominantly determined by area and height, whereas the second principal component was generally determined by features responsible for shape elongation (**Supplementary Table 3**). In the first main component of leaves, the greatest contribution was made by features that characterize the geometric size, such as perimeter, area, and height. The shape index, which is responsible for the leaf blade elongation, was predominantly a positive effector for the second principal component.

### 3.4 Machine learning algorithm - classification accuracy

The average values of morphological quantitative data for fruits, leaves and endocarps showed no adequate capacity to reliably discriminate olive cultivars according to Principal Component Analysis. In fact, discrimination between cultivars could be observed only if the morphological data of plant organs of individual plants was taken into consideration using multivariate statistical analysis.

**Figure 9:**
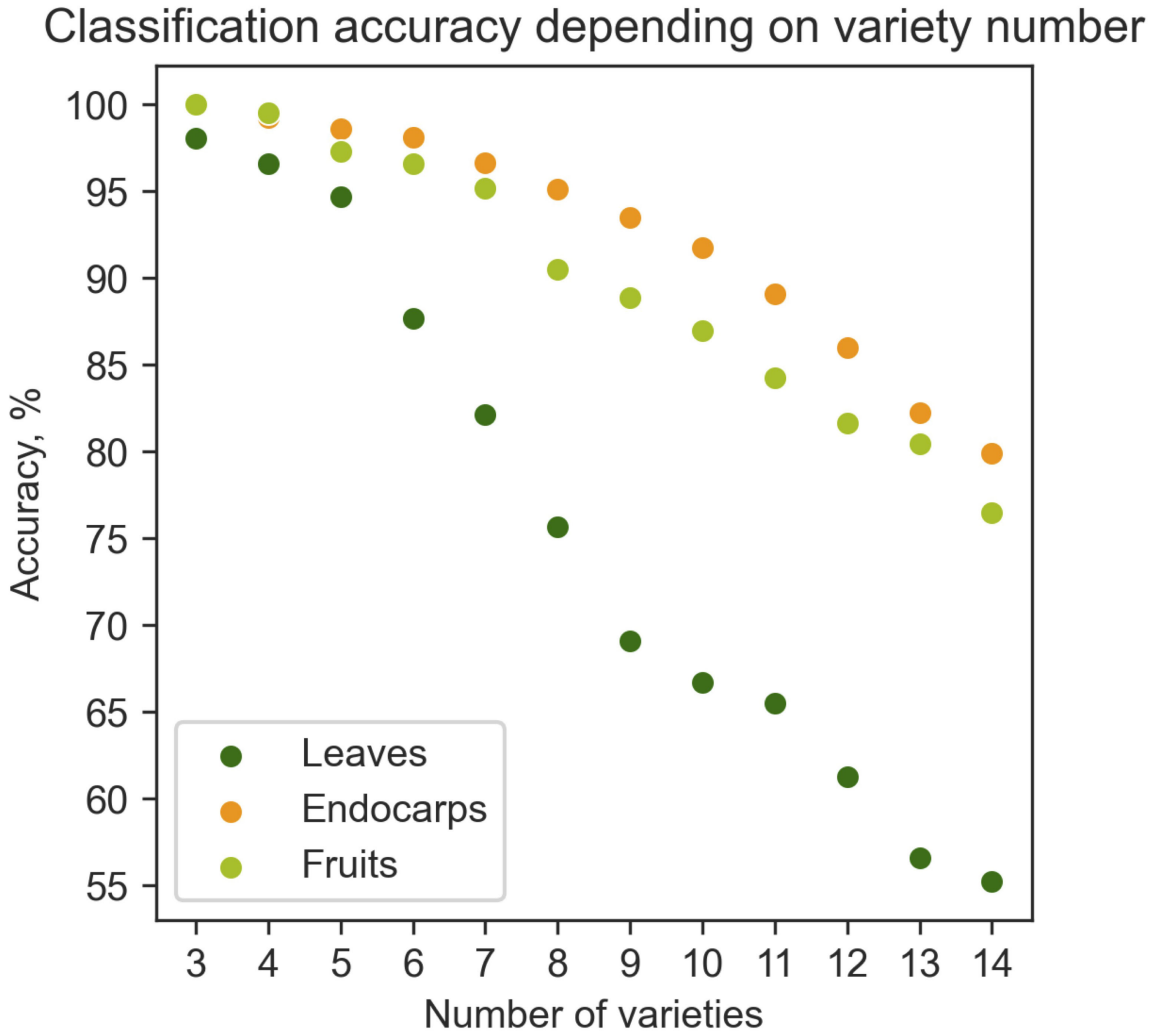
Dependence of classification accuracy on the number of olive cultivars. The fruit, endocarp and leaf morphological data were used to predict the percent accuracy of cultivar classification in relation to number of cultivars in the range of 3 to 14 cultivars.

Therefore, machine learning algorithms were used to classify and discriminate olive cultivars. The Extreme Gradient Boost (XGBoost) algorithm performed better than three other basic classifiers (Random Forest Classifier, k-Nearest Neighbor (ΚNN) and Support Vector Classifier (SVM)) (**Supplementary Table 4**). **Figure 9** illustrates the mean accuracy of the classifier with different numbers of cultivars. As expected, the number of olive cultivars determined the success of the classification algorithm. The higher the number of cultivars, the lower the classification accuracy (**Figure 9**). Up to 6 cultivars could be classified with higher than 85% accuracy, while 14 cultivars were classified with 76% and 80% accuracy using fruit and endocarp morphological data, respectively (**Figure 9**). The mean accuracy of leaf classifier was nearly 55%. These results indicate that endocarp morphological features have the greatest capacity for cultivar discrimination, since these traits are less affected by the variability of environmental conditions and the training systems.

Single classifiers can be used to classify data for relatively simple tasks. Olive cultivar discrimination is a complex task, and the combination of simple classifiers can significantly improve performance. A meta-classifier approach which uses three different combination methods (KNN, RandomForest, XGBoost) was implemented in a similar way to the combination of simple classifiers. This approach used the numerical data of the morphological features of fruits, leaves and endocarps. **Table 1** indicates the performance measures (precision, recall and F1-score values) of the meta-classifier approach on the data set. It is remarkable that the average accuracy of an identification was approximately 95%.

**Table 1:** Performance of meta-classifier.

	precision	recall	f1-score
Kothreiki	0.92 ± 0.07	0.97 ± 0.05	0.94 ± 0.05
Karidolia-chalkidikis	1.0 ± 0.0	1.0 ± 0.0	1.0 ± 0.0
Asprolia-alexandroupolis	0.95 ± 0.1	0.88 ± 0.13	0.91 ± 0.1
Arbequina	1.0 ± 0.0	0.95 ± 0.11	0.97 ± 0.07
Arbosana	0.98 ± 0.05	0.96 ± 0.07	0.96 ± 0.05
Megaron	0.95 ± 0.1	0.96 ± 0.08	0.95 ± 0.06
Tragolia	0.9 ± 0.13	0.93 ± 0.08	0.91 ± 0.08
Mavrolia-serron	0.94 ± 0.09	0.98 ± 0.05	0.96 ± 0.05
Koutsourelia	0.97 ± 0.04	0.98 ± 0.03	0.98 ± 0.03
Ntopia-atsiholou	0.93 ± 0.09	0.95 ± 0.06	0.94 ± 0.06
Kalamon	1.0 ± 0.0	1.0 ± 0.0	1.0 ± 0.0
Thiaki	0.97 ± 0.05	0.9 ± 0.09	0.93 ± 0.06
Mastoidis-gigas	0.97 ± 0.05	0.98 ± 0.04	0.97 ± 0.03
Koroneiki	0.92 ± 0.09	0.96 ± 0.04	0.94 ± 0.06
Total	0.96 ± 0.02	0.96 ± 0.02	0.95 ± 0.02

Performance measures using precision, recall and F1 score values.

### 3.5 Importance of each morphological feature and the contribution of plant organs to cultivar classification

Machine learning models are increasingly used to replace human decision-making. A concept from cooperative game theory could be used to detect the fair contribution of each morphological feature to olive cultivar identification. The importance of morphological features for classification by the XGBoost algorithm was analyzed on the entire dataset using Shapley values. A wider spread of Shapley values implies more differentiation in classification model output and therefore higher feature importance.

**Figure 10:**
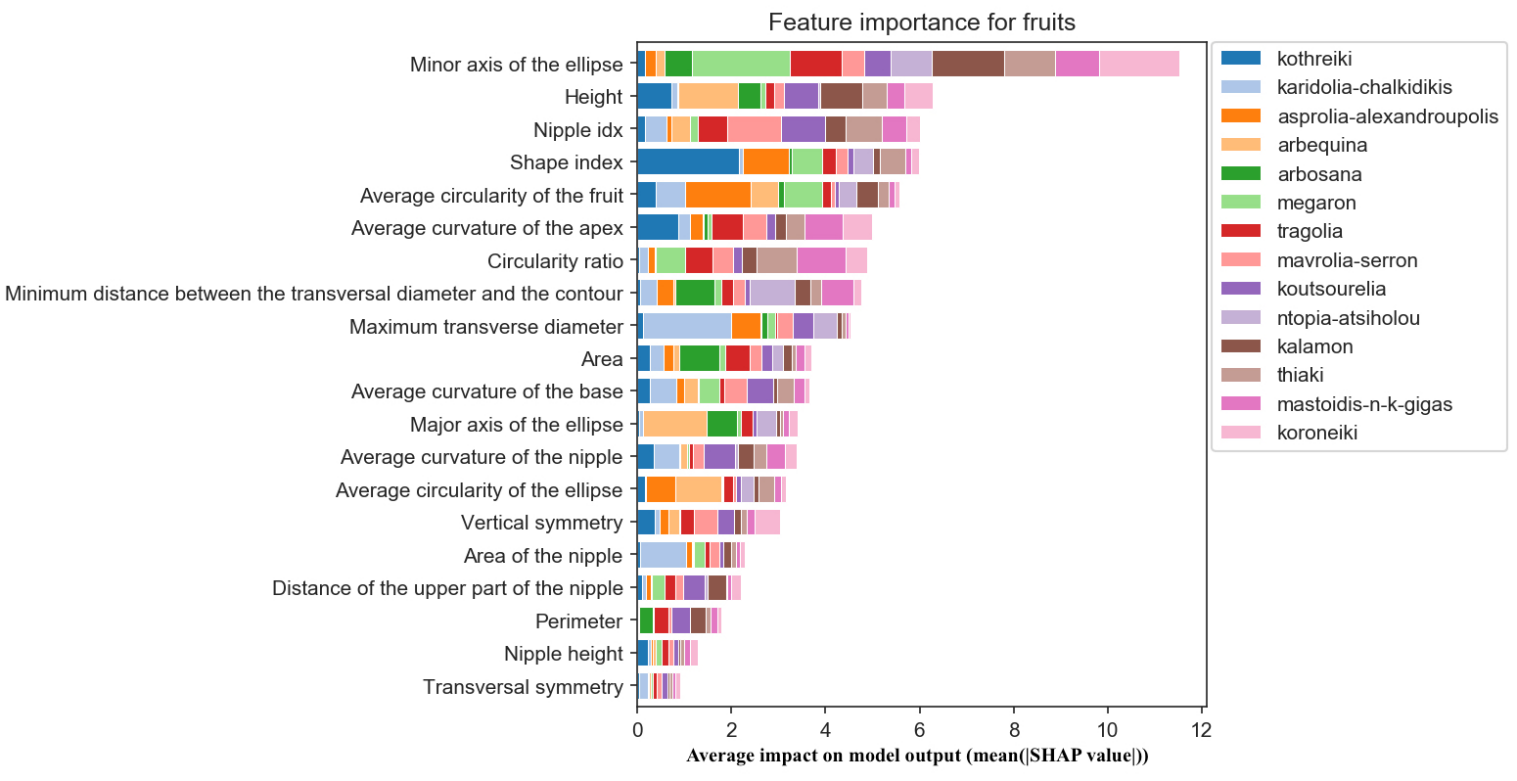
Morphological feature importance for fruits. The importance of each morphological parameter computed in olive fruit morphology.

As shown in **Figure 10**, the most important features for olive fruit classification were the minor axis of the ellipse and fruit height. The latter was followed by the presence or absence of a nipple, a feature which is commonly used in olive cultivar identification by visual assessment. However, it seemed that the measurements related to the nipple and the fruits’ transversal symmetry had little influence on the classification. The most important morphological features for the olive fruit classification of Kalamon are the height, the shape, the shape and size of a fitted ellipse (its curvature and the length of its major axis) and the fruit circularity (**Figure 10**). The most significant morphological characteristics for Karidolia Chalkidikis are the area, the size of a fitted ellipse (the length of its major and minor axes) and the position of the transversal diameter.

**Figure 11:**
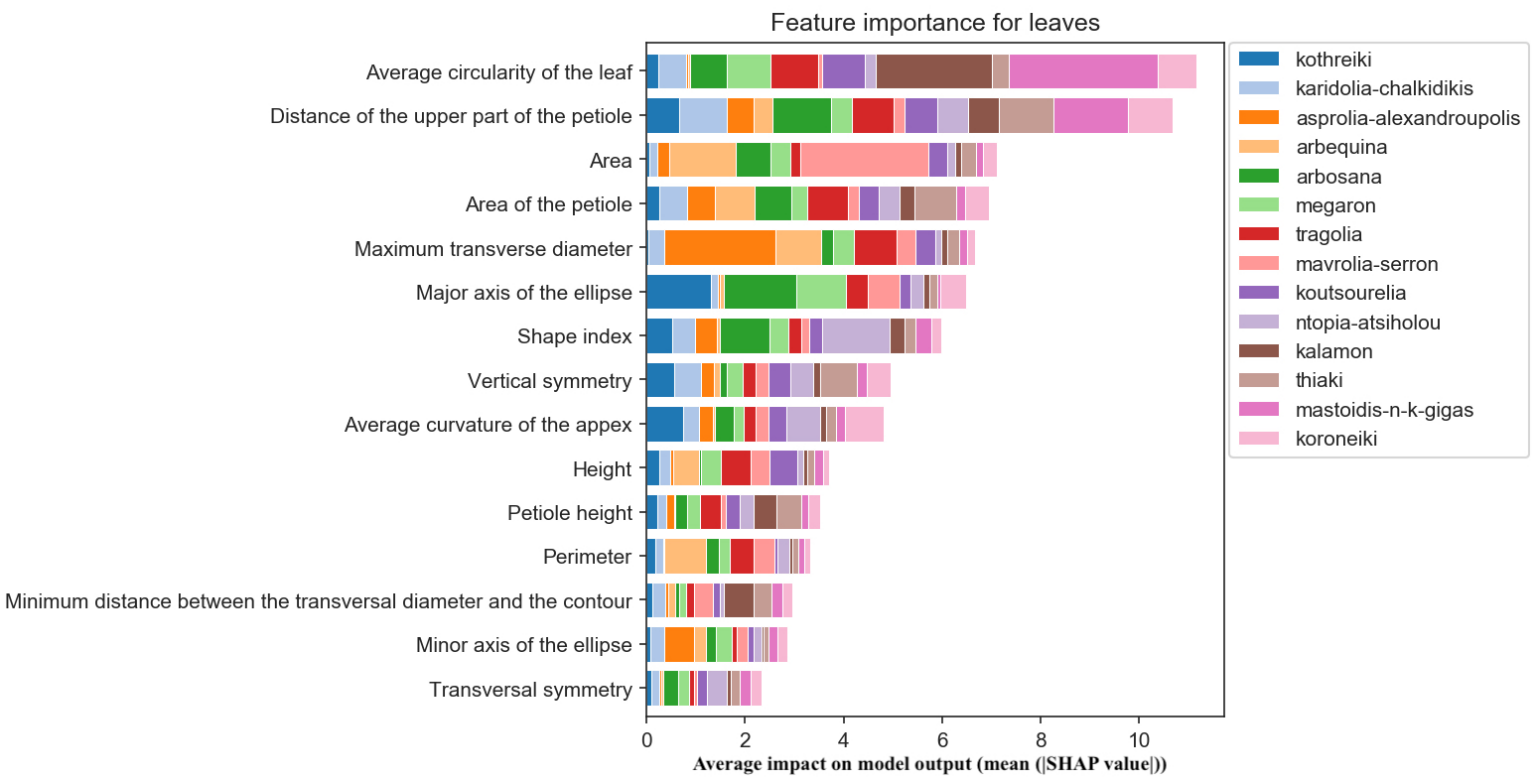
Morphological feature importance for leaves. The importance of each morphological parameter computed in olive leaf morphology.

The most important morphological feature for olive leaf classification was the roundness of the leaf blade, which is important for the identification of Megaron, the cultivar with the narrowest leaves, and Thiaki, the cultivar with the most circular leaves. The thickness of the petiole was also of importance for olive cultivar identification (**Figure 11**).

**Figure 12:**
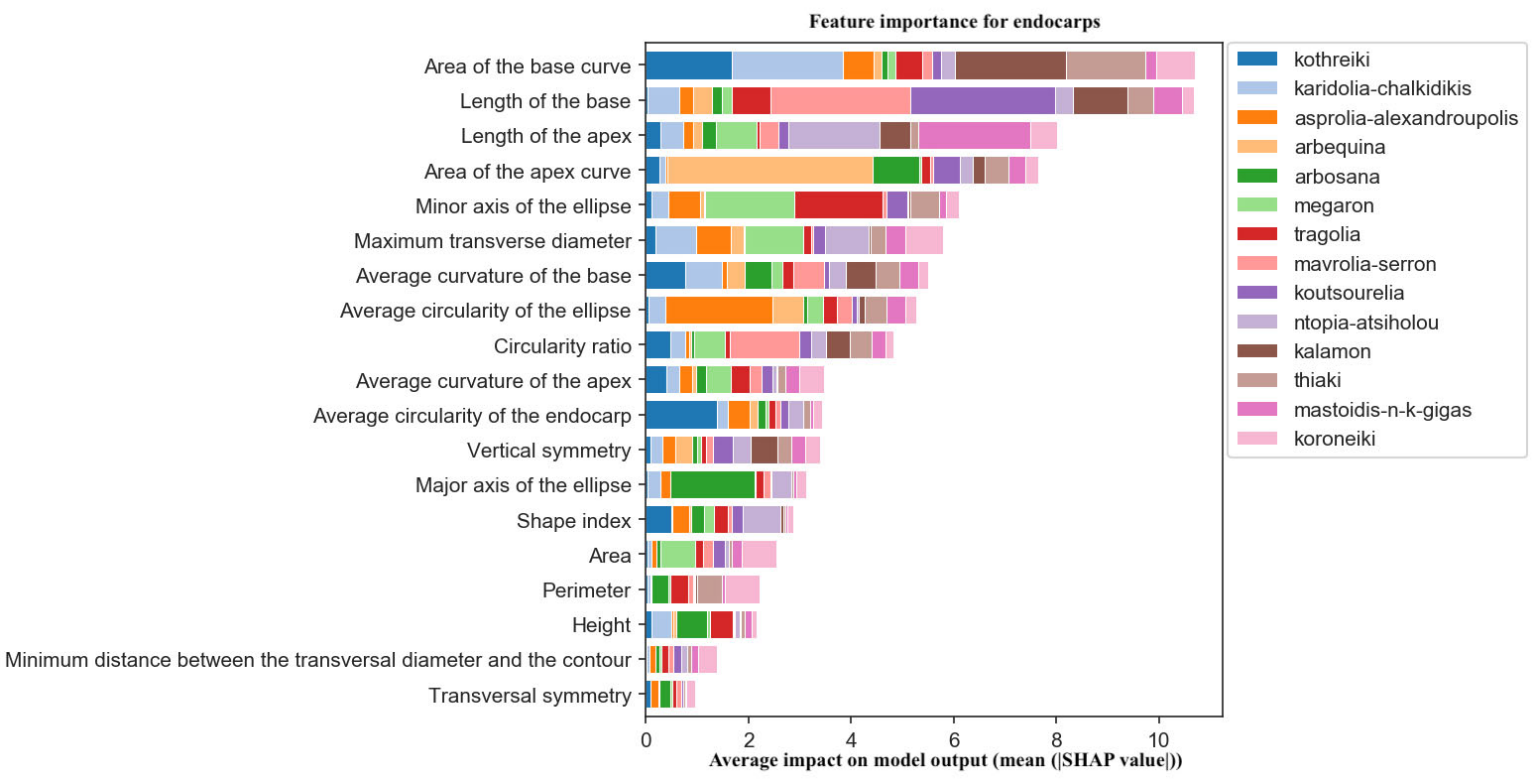
Morphological feature importance for endocarps. The importance of each morphological parameter computed in olive fruit endocarp morphology.

For endocarp classification, the key features were the area and the length of the base, which are related to the presence of a mucro. This trait is widely used for the identification of olive cultivars. Cultivars with shortened or elongated apexes and bases, such as Koutsourelia, Mastoidis, Arbequina and Arbosana, were also efficiently discriminated by the algorithm (**Figure 12**).

**Figure 13:**
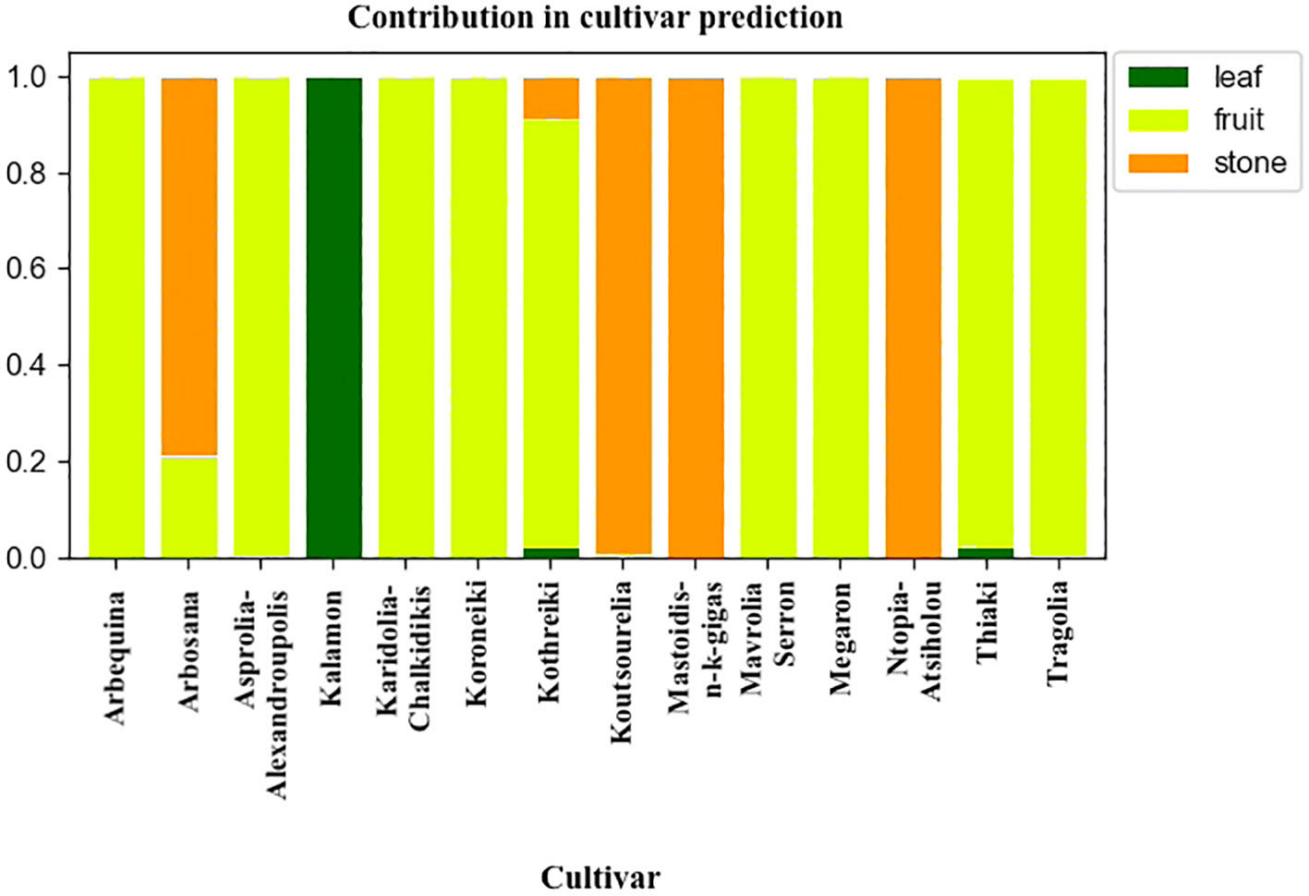
Plant organ contribution to cultivar identification for each cultivar.

The analysis of the characteristics of the meta-classifier revealed the contribution of either fruit, leaf or endocarp in the classification process. **Figure 13** depicts the contribution of each plant organ in cultivar identification. The discrimination of most cultivars was guided by both olive endocarps and fruits. The leaves only contributed to the identification of the Kalamon cultivar, the olive cultivar with the largest leaf blade.

## 4 Discussion

Morphological characterization has been used primarily to assess the diversity of olive genetic resources (Beiki et al., 2012; Trujillo et al., 2014) and to correctly identify and discriminate cultivars. Synonymous cultivars corresponding to different genotypes or identical cultivars with different names are frequently observed in germplasm collections (Ganino et al., 2006). In addition, cultivar identification is used extensively for food authentication purposes to ensure that the cultivar origin on an olive oil label correspond to the bottled product (Trujillo et al., 2014). Moreover, the sorting of olive fruits according to cultivar origin before milling and of table olives according to cultivar and fruit size and shape are important for the food industry (Puerto et al., 2015; Beyaz et al., 2017; Ponce et al., 2018, Ponce et al., 2019; Gomes et al., 2020; Khadivi et al., 2022; Gago et al., 2024).

Efficient morphological characterization requires the determination of morphological characteristics including fundamental shape descriptors of plant organs such as fruits, leaves and endocarps by using semi-automatic methodologies in a numerically precise manner (Blazakis et al., 2017) to increase the reliability of the measurements.

The variation in environmental conditions causes variation in the morphological characteristics. This variation was analyzed in 14 olive cultivars using split violin plots in order to determine which morphological characteristics were less affected by environmental factors. The presence of the nipple and the shape index of fruit were marginally affected by the environmental conditions, therefore remained stable for all cultivars. Morphological characteristics related to fruit size, such as perimeter and area, showed high variability between the two growing periods. The morphological analysis of leaves showed that the circularity remained stable in the two growing periods, whereas the transversal symmetry of the leaf blade varied greatly. These results indicate that there are morphological characteristics which are stable and are not affected by the environmental conditions in each growing period suggesting that are mainly defined by genetic factors. These traits might be considered of particular importance for breeding purposes since they can be used as specific morphological markers to identify specific cultivars.

The numerical data from morphological analysis were processed using principal component analysis for olive cultivar identification. This approach did not discriminate cultivars indicating that the use of multivariate statistical techniques with principal component analysis was not efficient. In another study, a versatile algorithm was used for discriminative variable selection as an additional methodology along with principal component analysis (Vanloot et al., 2014). An attempt by using the multivariate statistical method “Orthogonal Partial Least Square Discriminant Analysis” managed to discriminate six Greek protected designation of origin table olive varieties with almost 98% correct classification using morphological characteristics (Agriopoulou et al., 2021).

The use of morphological characteristics from either only fruits or leaves or endocarps resulted in discriminatory capacity of lower efficiency (Orrù et al., 2013; Fuentes et al., 2018). This suggested the importance of determining the most important characteristics for classification of olive cultivars, as well as the need to consider different classification algorithms. The use of basic classifiers to discriminate cultivars by taking into consideration only quantitative morphological data revealed that olive fruits and endocarps resulted in better classification accuracy than leaves. Moreover, the endocarp was the most suitable organ for cultivar identification because it showed lower variation than fruit and leaf in all morphological characteristics in two growing periods. This might be attributed to the wooden origin of the endocarp, the protection by the olive mesocarp pulp, and the lower impact of climatic conditions on its shape features (Trujillo et al., 2014; Koubouris et al., 2019). However, a previous study employed a computer-image analysis approach to classify olive cultivars using mathematical tools, specifically fractal geometry and moments (Bari et al., 2003). The methodology focused on analyzing the surface and shape characteristics for olive cultivar identification based on the endocarps of nine different cultivars leading to the classification of approximately 55% of the cultivars (Bari et al., 2003).

The combinatorial approach of olive leaf, fruit, and endocarp classifiers showed greater potential compared to individual classifiers, since the final prediction of the cultivar was determined by the organ with numerical traits exhibiting the highest discriminatory power. Analysis of the meta-classifier traits for each cultivar indicated which plant organ and trait showed the highest discriminatory power. This approach showed a very high classification accuracy among 14 olive cultivars in comparison to previous studies. The meta-classifier approach revealed morphological traits with higher discriminatory power, which are not always those with lower variability in the two growing seasons. This is because it considers the combined data of olive leaf, fruit, and endocarp morphological analysis. Accurate measurements of the most characteristic plant organs would be particularly suitable for inputs into models of genetic selection which are built on the concept of quantitative trait loci (QTLs) (Sadok et al., 2013; Kaya et al., 2019). Moreover, the minor axis of the ellipse, the height, the nipple index and the shape index were determined as the most important morphological characteristics of fruit for cultivar discrimination while the least important was the transversal symmetry. Taking into consideration these data, a strategy can be developed to prioritize morphological characteristics for cultivar identification purposes in order to accelerate the whole process for a large number of fruit samples.

Automated cultivar identification of olives and other species using morphological characteristics is a challenging task (Ponce et al., 2019). Artificial neural networks have been used mostly for specific olive cultivar identification cases in which the statistical standards proved to be insufficient. An attempt was made previously to use traits related to leaf morphologies in an artificial neural network methodology to discriminate different olive cultivars (Mancuso and Nicese, 1999). In another report, Artificial Neural Network analysis was utilized to assess data on the length, width, and color of the fruits and endocarps (Beyaz et al., 2017). Sets of five and seven cultivars were correctly identified with an accuracy of 89% and 90%, respectively (Beyaz et al., 2017). In addition, partial least squares-discriminant analysis (PLS-DA) based on morphological characteristics and texture features of olive endocarps resulted in 89% accurate classification of a set of five Spanish cultivars (Martínez et al., 2018) while a similar methodology on morphological and chemometric analysis from imaging data using different endocarp positions achieved 100% correct classification of five French cultivars in an approach similar to the one used in this report (Vanloot et al., 2014). Moreover, different artificial neural network models were used with commercial software to assess their use in the identification of a set of eleven Spanish and Turkish cultivars, utilizing the color properties, length and width of an olive fruit and stone (Beyaz and Öztürk, 2017). In that case, olive cultivars were classified with more than 91% accuracy.

The current study demonstrates that morphological markers, specifically the geometrical features of fruits, leaves, and endocarps, could effectively identify and discriminate olive germplasm. By employing machine learning techniques and a meta-classifier approach, the methodology achieved a 95% accuracy rate in classifying 14 olive cultivars over two consecutive growing periods. The study quantified the contribution of each morphological feature to the discrimination process, highlighting that endocarps and fruits were essential for identifying most cultivars, while leaves were particularly significant for identifying the Kalamon cultivar. The combined use of morphological traits from these three olive organs enhanced the overall discrimination capacity, suggesting the methodology’s potential application in olive breeding and cultivar identification.

Machine learning algorithms were used for cultivar classification and other applications (Fuentes et al., 2018; Ishikawa et al., 2018; Miho et al., 2024). A statistical methodology was developed based on classification binary trees for the discrimination of different morphologies of endocarps (Koubouris et al., 2019). A powerful image processing, artificial intelligence approach was used by designing a procedure based on convolutional neural networks (CNN) and image processing to classify images of olive fruits (Ponce et al., 2019). In this report, the classification accuracy for the discrimination of up to 8 cultivars was approximately 90% by using the basic classifier. However, the classification accuracy for the discrimination of 14 cultivars was higher than 95% by using the meta-classifier approach. These results indicate that the use of a meta-classifier combined with the quantitative data of endocarp, fruit and leaf morphological characteristics has higher capacity for discrimination.

The proposed methodology discriminated higher number of olive cultivars compared to the other approaches (Beyaz et al., 2017; Fuentes et al., 2018; Ishikawa et al., 2018; Koubouris et al., 2019; Ponce et al., 2019; Agriopoulou et al., 2021). It is comprised of an integrated method to morphological description and discrimination of olive cultivars that uses image analysis tools and machine learning algorithms. While machine learning is a data analytics technique that teaches computational approaches to “learn” information directly from data without relying on a predetermined model, the algorithms adaptively improve their performance as the number of samples available for learning increases (Guide, 1998). One limitation of the current approach is the lack of data related to color traits of olive fruits, leaves and endocarps. Moreover, the depth and the pattern of grooves of endocarps, valuable characteristics for cultivar identification, were not taken into consideration (Trujillo et al., 2014). In the future, additional qualitative measurements of attributes regarding the texture and color of olive fruit, leaf or endocarp could be considered, increasing the classification accuracy of the current approach. This methodology is based on two-dimensional morphological data on olive cultivars, but a focus on three-dimensional settings might be the next step. However, this approach requires additional infrastructure and introduces different computational challenges.

## 5 Conclusion

The discrimination of olive cultivars by using morphological characteristics of their fruits, leaves and endocarps was performed up to now mostly by visual assessment and was considered challenging to transfer the classification of the visual assessment to an automated computerized methodology. This report suggest that machine learning is efficient and accurate for the classification of olive cultivars, while the algorithms provide information on the contribution of fruit, leaf and endocarp morphological characteristics to the discrimination process. Finally, the proposed methodology has the capacity to provide the basis for a wide range of applications for olive cultivar identification including breeding.

## Data availability statement

The raw data supporting the conclusions of this article will be made available by the authors, without undue reservation.

## Author contributions

PK: Conceptualization, Formal analysis, Funding acquisition, Investigation, Methodology, Supervision, Writing – original draft, Writing – review & editing. KB: Data curation, Formal analysis, Methodology, Software, Writing – original draft, Writing – review & editing. DS: Data curation, Formal analysis, Methodology, Writing – original draft, Writing – review & editing. MK: Data curation, Writing – original draft, Writing – review & editing. ME: Data curation, Writing – original draft, Writing – review & editing. AA: Writing – original draft, Writing – review & editing. GK: Data curation, Methodology, Writing – original draft, Writing – review & editing.
