# Supplementary material for: Discrimination of 14 olive cultivars using morphological analysis and machine learning algorithms
Source: Front Plant Sci. 2024 Aug 8;15:1441737. doi: 10.3389/fpls.2024.1441737 (PMC11340652; doi:10.3389/fpls.2024.1441737)

## Slide 1
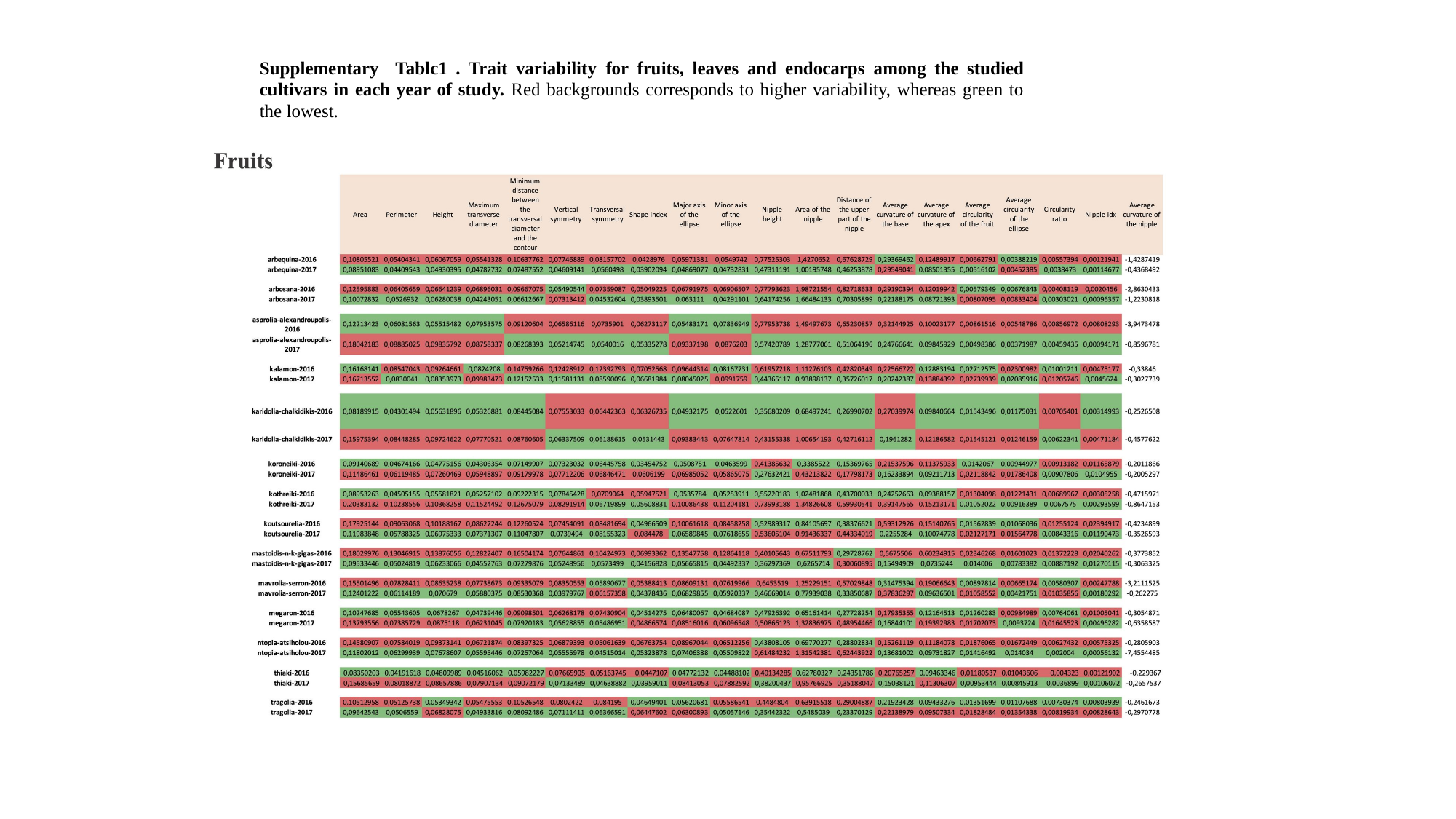

Supplementary Tablc1 . Trait variability for fruits, leaves and endocarps among the studied cultivars in each year of study. Red backgrounds corresponds to higher variability, whereas green to the lowest.

## Slide 2
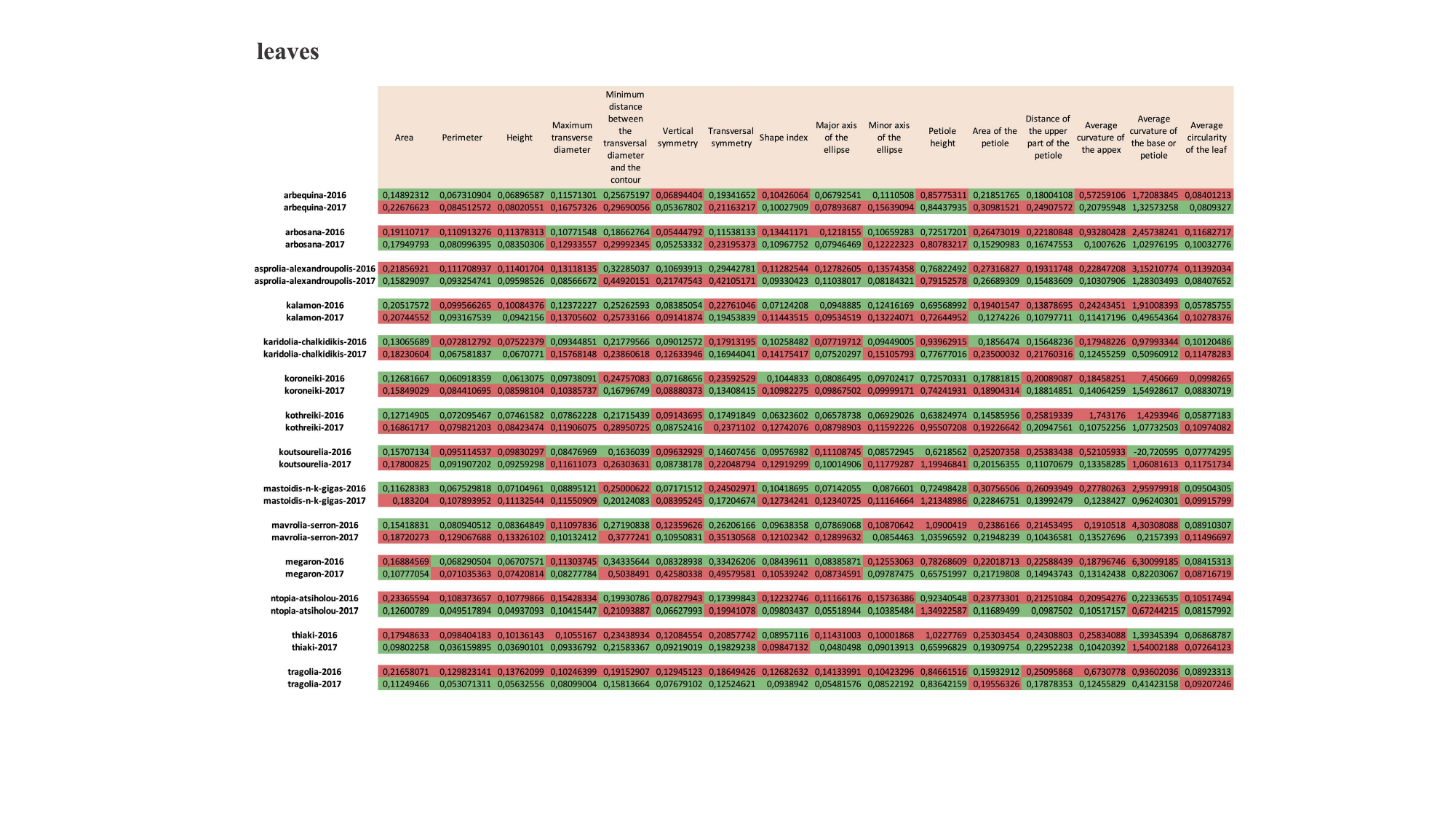

## Slide 3
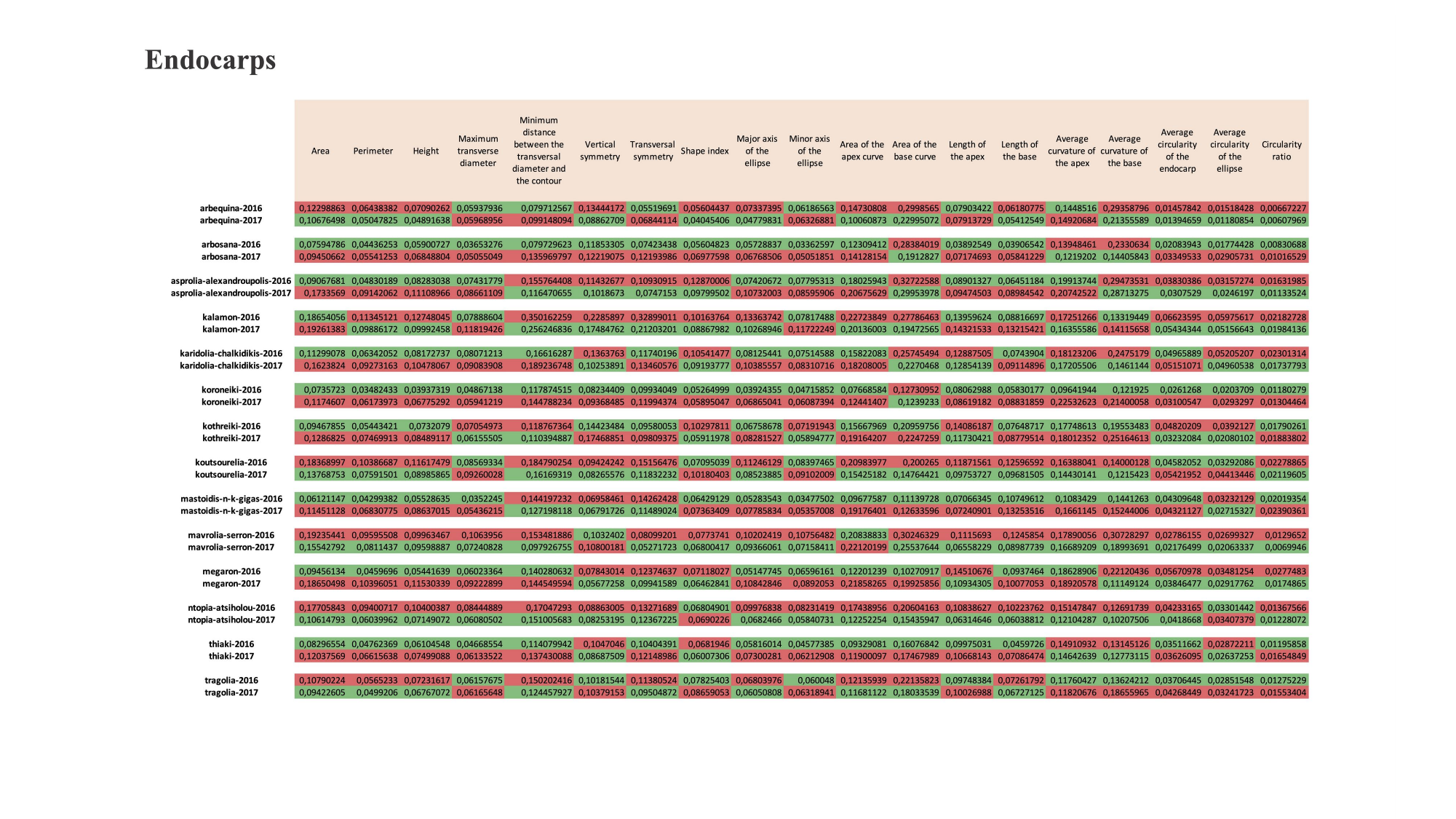

Supplement: Supplementary file 1 [file Presentation_1.pptx]
