# Supplementary material for: Discrimination of 14 olive cultivars using morphological analysis and machine learning algorithms
Source: Front Plant Sci. 2024 Aug 8;15:1441737. doi: 10.3389/fpls.2024.1441737 (PMC11340652; doi:10.3389/fpls.2024.1441737)

## Slide 1
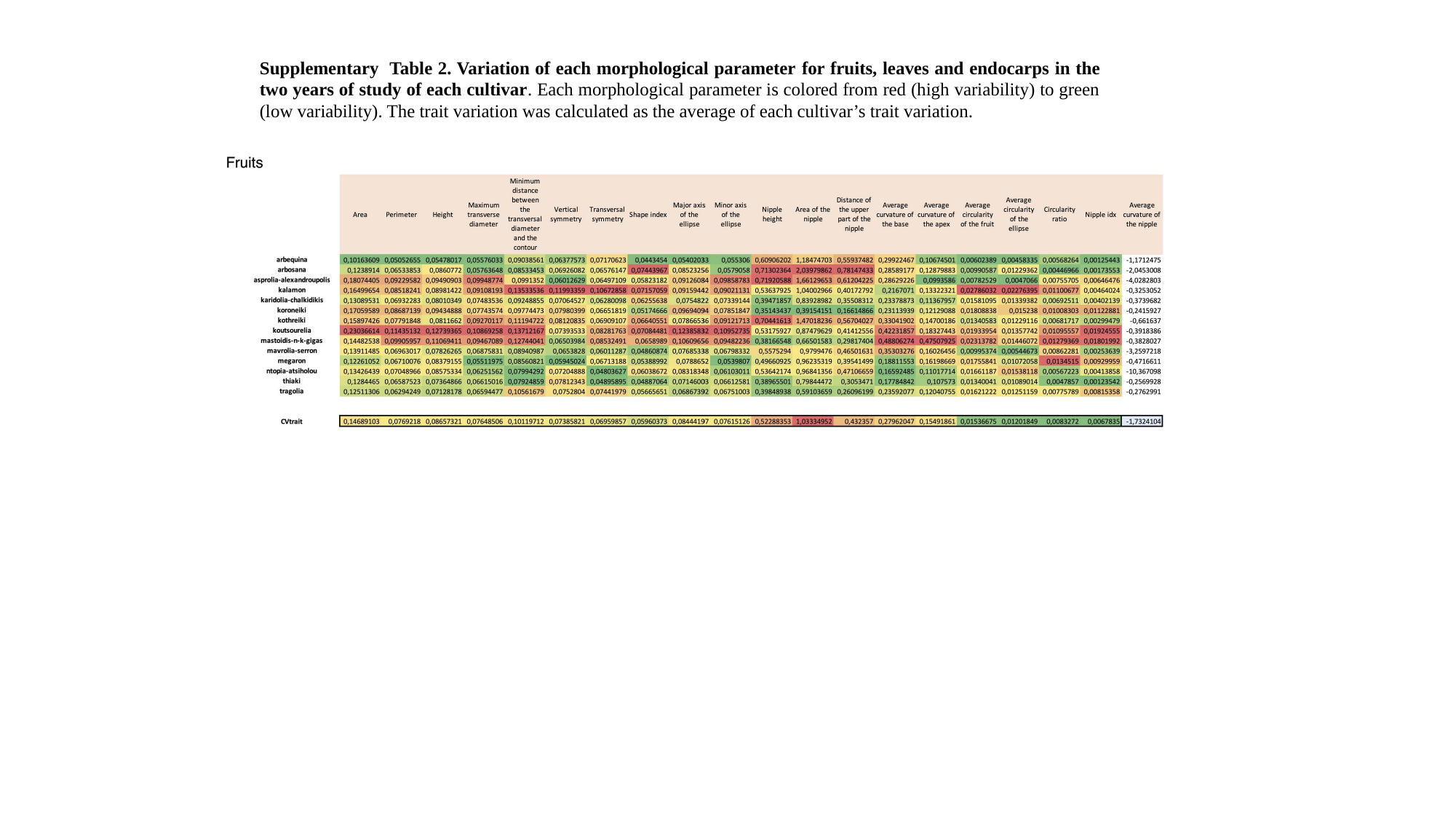

Supplementary Table 2. Variation of each morphological parameter for fruits, leaves and endocarps in the two years of study of each cultivar. Each morphological parameter is colored from red (high variability) to green (low variability). The trait variation was calculated as the average of each cultivar’s trait variation.

## Slide 2
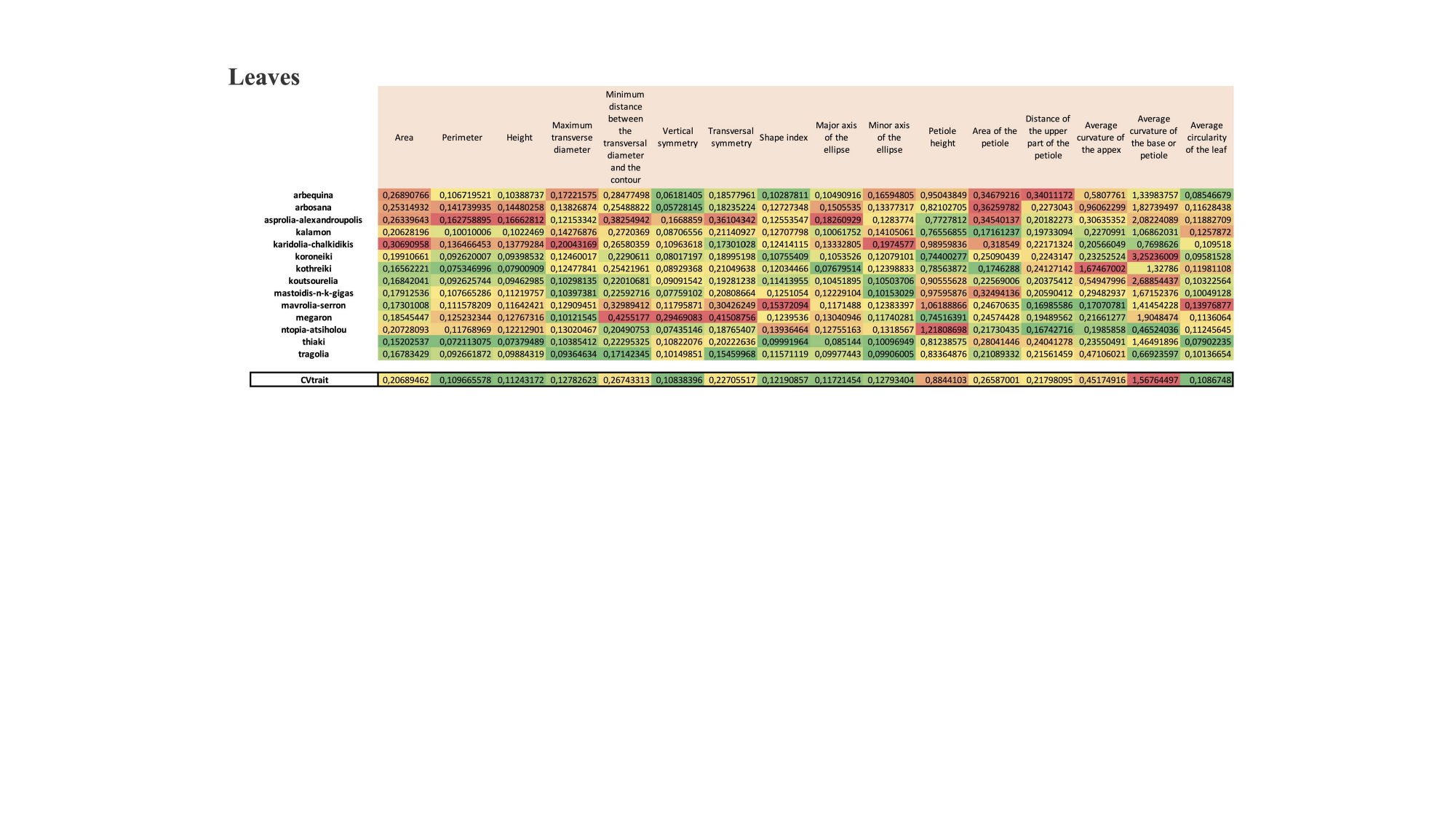

## Slide 3
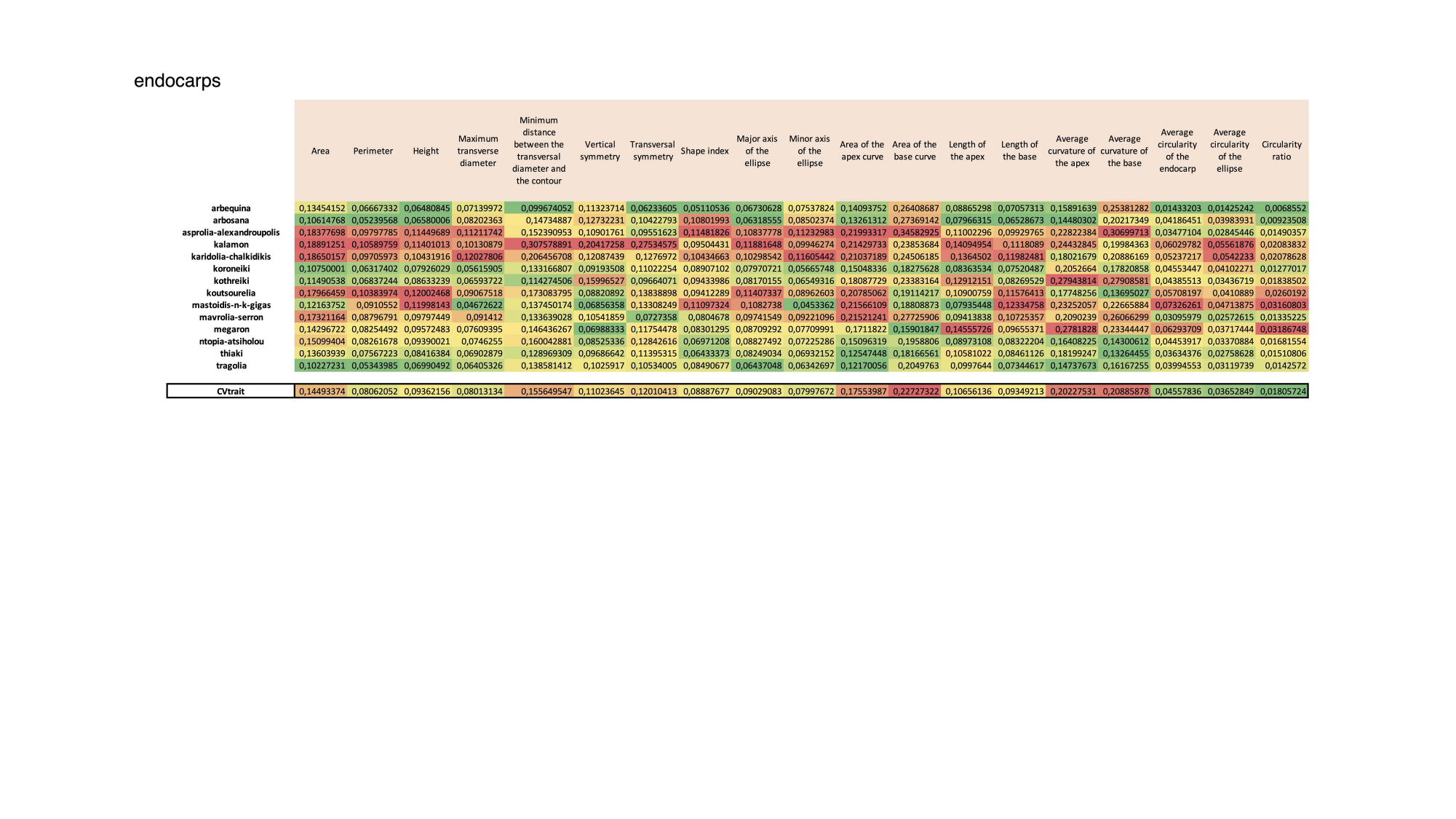

Supplement: Supplementary file 2 [file Presentation_2.pptx]
