# Supplementary material for: Discrimination of 14 olive cultivars using morphological analysis and machine learning algorithms
Source: Front Plant Sci. 2024 Aug 8;15:1441737. doi: 10.3389/fpls.2024.1441737 (PMC11340652; doi:10.3389/fpls.2024.1441737)
Supplement: Supplementary file 3 [file Table_1.docx]

Supplementary Material

**Supplementary Figure 1.** Meta classifier training scheme. At the beginning, three independent base classifiers are trained: for leaves, fruits, and endocarps. The output is probabilities belonging to varieties. Then these probabilities are used to train the meta-classifier.
