# Supplementary material for: Discrimination of 14 olive cultivars using morphological analysis and machine learning algorithms
Source: Front Plant Sci. 2024 Aug 8;15:1441737. doi: 10.3389/fpls.2024.1441737 (PMC11340652; doi:10.3389/fpls.2024.1441737)
Supplement: Supplementary file 4 [file Table_2.docx]

Supplementary Material


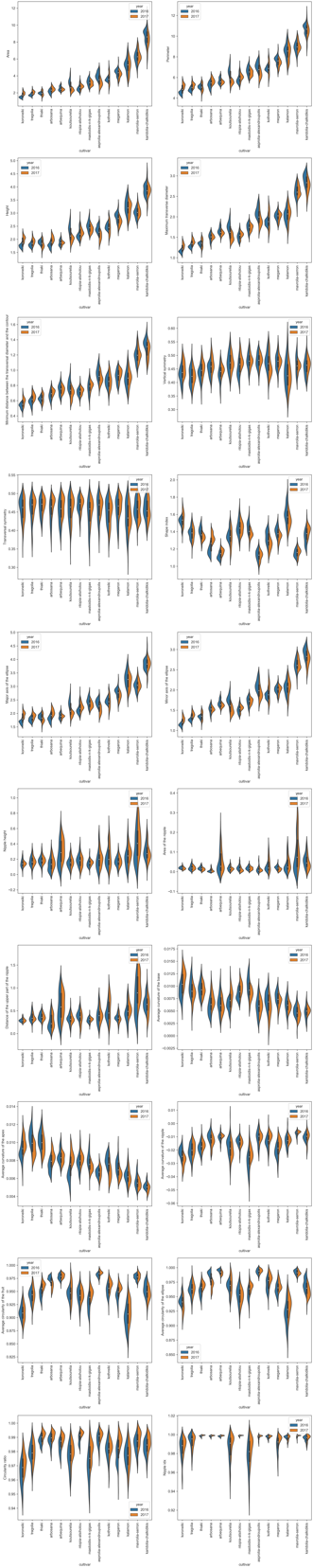

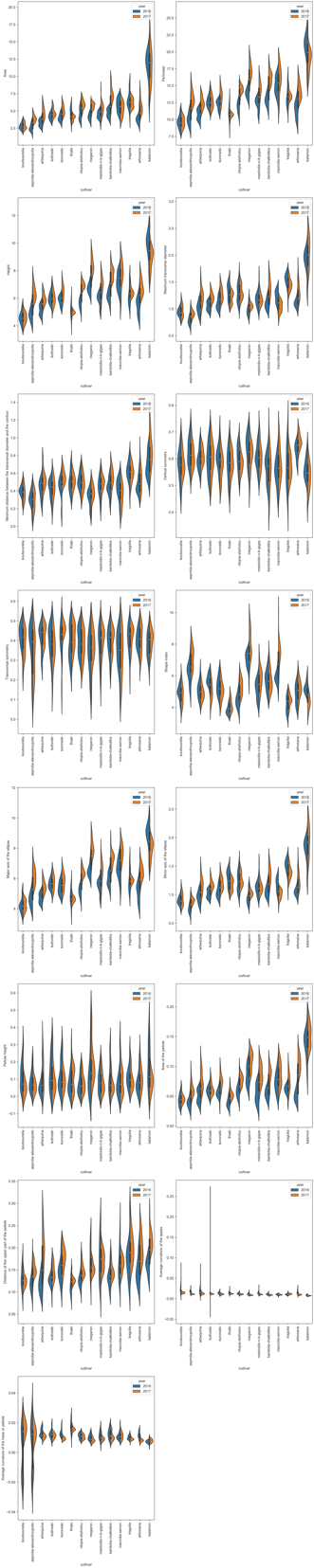

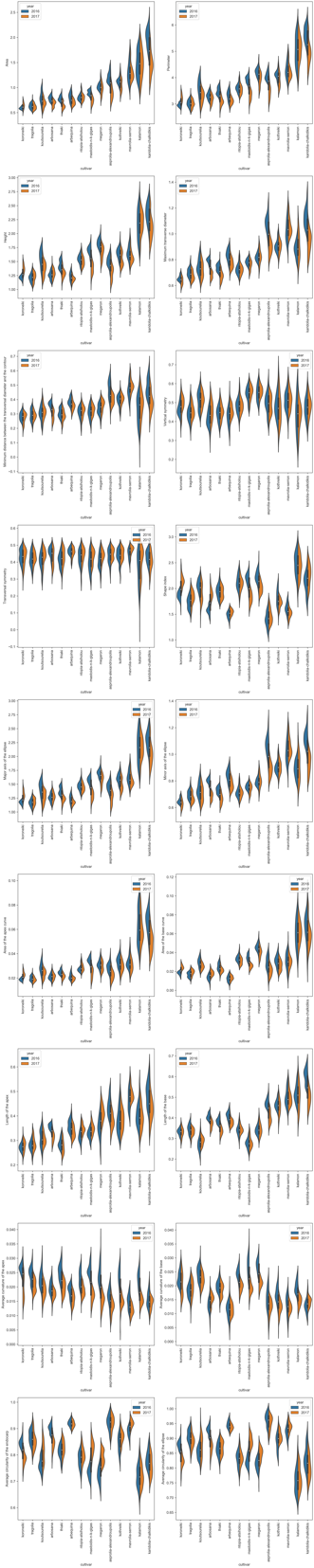


**Supplementary Figure 2.** Split violin plots for the fruits, leaves and endocarps morphological parameters.
