# Supplementary material for: Discrimination of 14 olive cultivars using morphological analysis and machine learning algorithms
Source: Front Plant Sci. 2024 Aug 8;15:1441737. doi: 10.3389/fpls.2024.1441737 (PMC11340652; doi:10.3389/fpls.2024.1441737)
Supplement: Supplementary file 5 [file Table_3.docx]

Supplementary Material

**Supplementary Table 3**: Factor loadings for each variable on the components of PCA analysis of fruits, leaves and endocarps.

| Fruits | | |
| --- | --- | --- |
|  | PC1 | PC2 |
| Average curvature of the apex | -0.27 | -0.08 |
| Average curvature of the base | -0.23 | 0.13 |
| Shape index | -0.11 | 0.41 |
| Transversal symmetry | -0.01 | -0.12 |
| Vertical symmetry | 0.05 | -0.00 |
| Circularity ratio | 0.08 | -0.31 |
| Nipple idx | 0.08 | -0.26 |
| Average circularity of the ellipse | 0.10 | -0.41 |
| Average circularity of the fruit | 0.11 | -0.44 |
| Area of the nipple | 0.18 | -0.06 |
| Nipple height | 0.19 | -0.11 |
| Average curvature of the nipple | 0.20 | -0.26 |
| Distance of the upper part of the nipple | 0.22 | -0.11 |
| Height | 0.28 | 0.25 |
| Major axis of the ellipse | 0.29 | 0.24 |
| Perimeter | 0.31 | 0.17 |
| Area | 0.31 | 0.15 |
| Minimum distance between the transversal diameter and the contour | 0.32 | 0.03 |
| Maximum transverse diameter | 0.33 | 0.06 |
| Minor axis of the ellipse | 0.33 | 0.06 |
| Leaves | | |
|  | PC1 | PC2 |
| Average curvature of the appex | -0.25 | -0.14 |
| Shape index | -0.08 | 0.53 |
| Vertical symmetry | -0.05 | 0.12 |
| Transversal symmetry | 0.06 | -0.24 |
| Average circularity of the leaf | 0.06 | -0.53 |
| Petiole height | 0.08 | 0.04 |
| Distance of the upper part of the petiole | 0.24 | -0.06 |
| Minimum distance between the transversal diameter and the contour | 0.29 | -0.27 |
| Major axis of the ellipse | 0.31 | 0.28 |
| Height | 0.33 | 0.24 |
| Area of the petiole | 0.33 | 0.12 |
| Perimeter | 0.33 | 0.21 |
| Minor axis of the ellipse | 0.34 | -0.18 |
| Maximum transverse diameter | 0.34 | -0.18 |
| Area | 0.35 | -0.01 |
| Endocarps | | |
|  | PC1 | PC2 |
| Average curvature of the apex | -0.13 | -0.23 |
| Average circularity of the ellipse | -0.13 | 0.36 |
| Average circularity of the endocarp | -0.12 | 0.38 |
| Average curvature of the base | -0.09 | -0.31 |
| Circularity ratio | -0.04 | 0.27 |
| Transversal symmetry | -0.03 | 0.20 |
| Vertical symmetry | 0.02 | -0.08 |
| Shape index | 0.12 | -0.37 |
| Minimum distance between the transversal diameter and the contour | 0.19 | 0.28 |
| Length of the apex | 0.26 | 0.18 |
| Length of the base | 0.27 | 0.17 |
| Minor axis of the ellipse | 0.27 | 0.22 |
| Maximum transverse diameter | 0.27 | 0.21 |
| Area of the base curve | 0.29 | -0.17 |
| Area of the apex curve | 0.31 | -0.11 |
| Height | 0.31 | -0.14 |
| Major axis of the ellipse | 0.32 | -0.12 |
| Area | 0.33 | 0.04 |
| Perimeter | 0.33 | -0.05 |
