# Supplementary material for: Discrimination of 14 olive cultivars using morphological analysis and machine learning algorithms
Source: Front Plant Sci. 2024 Aug 8;15:1441737. doi: 10.3389/fpls.2024.1441737 (PMC11340652; doi:10.3389/fpls.2024.1441737)
Supplement: Supplementary file 6 [file Table_4.docx]

Supplementary Material

**Supplementary Table 4**: Mean and standard deviation F1-score by 10-fold cross-validation for 14 cultivars by different algorithms

| **Organ** | **Algorithm** | **Score** |
| --- | --- | --- |
| **Fruits** | **XGBoost** | 80.0 ± 5.0 |
|  | **RandomForest** | 72.7 ± 5.0 |
|  | **KNN** | 66.7 ± 4.7 |
|  | **SVC** | 64.9 ± 4.5 |
| **Leaves** | **XGBoost** | 55.4 ± 8.8 |
|  | **RandomForest** | 48.3 ± 6.5 |
|  | **KNN** | 40.0 ± 5.5 |
|  | **SVC** | 44.9 ± 7.5 |
| **Endocarps** | **XGBoost** | 78.5 ± 3.8 |
|  | **RandomForest** | 68.0 ± 7.1 |
|  | **KNN** | 60.8 ± 7.4 |
|  | **SVC** | 61.9 ± 8.4 |
